# Protein kinase CK2α’ as a dual modulator of neuroimmune signaling and synaptic dysfunction in Tauopathy

**DOI:** 10.21203/rs.3.rs-7078069/v1

**Published:** 2025-08-07

**Authors:** Angel White, Peter Gavrilyuk, Rafael Falcon Moya, Reid Thurston, Amal Fickak, Nicholas B Rozema, Prarthana Keshavaram, Scott Vermilyea, Riley Schlichte, Joyce Meints, Ying Zhang, Alfonso Araque, Michael Lee, Rocio Gomez-Pastor

**Affiliations:** University of Minnesota

**Keywords:** Tauopathy, Synaptic function, Microglia, CK2, CK2α’

## Abstract

**Background:**

Tauopathies are a group of neurodegenerative diseases characterized by tau accumulation, neuroinflammation, and synaptic dysfunction, yet effective treatments remain elusive. Protein Kinase CK2 has been previously associated with different aspects of tau pathology but genetic evidence for the contribution of CK2 to tauopathy remained lacking.

**Methods:**

We used cell and mouse models to explore the impact of CK2α’ in tauopathy. We explored our hypothesis using molecular, biochemical, behavioral and electrophysiological techniques.

**Results:**

Here, we show CK2α’, one of the two catalytic subunits of CK2, as a novel regulator of tau-mediated neurodegeneration. We found that CK2α’ expression is elevated in the hippocampus of PS19 tauopathy mice and in postmortem brains of dementia patients, particularly in neurons and microglia. Using genetic haploinsufficiency in PS19 mice, we demonstrated that reduced CK2α’ levels significantly decrease phosphorylated tau and total tau burden in the hippocampus and cortex. CK2α’ depletion also enhanced synaptic gene expression, synaptic density, and LTP, while attenuating microglial activation, synaptic engulfment, and pro-inflammatory cytokine levels. Importantly, CK2α’ depletion rescued cognitive deficits assessed in the Barnes maze. These effects appear to be mediated through both neuronal and glial functions and may involve CK2α’-dependent modulation of tau-associated phosphorylation and neuroinflammatory and immune signaling pathways.

**Conclusions:**

Our findings highlight CK2α’ as a key node at the intersection of tau pathology, synaptic dysfunction, and neuroimmune signaling. Targeting CK2α’ may offer a novel and selective therapeutic strategy for modifying disease progression in tauopathies.

## Background

Tauopathies encompass a broad range of neurodegenerative conditions, most notably Alzheimer’s disease (AD) and related dementias (ADRD), for which no cure currently exists [1-3]. These disorders are generally categorized as either primary or secondary tauopathies. Primary tauopathies result from direct mutations in the microtubule-associated protein tau (MAPT), a protein critical for microtubule stabilization and intracellular transport [4, 5]. In contrast, secondary tauopathies arise from pathological tau accumulation triggered by mutations in genes other than MAPT [6]. Regardless of the cause, tauopathies are typically marked by progressive neuronal loss, synaptic and cognitive dysfunction, and widespread inflammation in both the central nervous system and peripheral tissues [7-11]. A deeper understanding of the molecular mechanisms underlying tauopathies and their downstream consequences is essential for developing effective disease-modifying therapies. One key area of research is the role neuroinflammation plays in the progression of tauopathies. These disorders are characterized by chronic, excessive inflammatory responses to abnormal tau proteins [8, 9, 12]. This persistent inflammation can exacerbate tau pathology by promoting sustained neuronal damage and dysfunction [12-15]. However, despite ongoing research, the mechanisms that connect neuroinflammation and tau pathology are still poorly understood.

Protein kinase CK2 (formerly known as Casein Kinase II), is a serine/threonine kinase that has emerged as an important regulator of neuroinflammation and protein homeostasis in several neurodegenerative diseases [16-18], and it has previously been connected to tau pathology in AD[19-22]. However, its specific role in the inflammatory pathways associated with tauopathies remains poorly defined. Interestingly, CK2 has demonstrated potential as a therapeutic target in various neurodegenerative contexts [17, 18]. In AD, CK2 has been implicated in processes such as synaptic plasticity [23, 24], amyloid precursor protein (APP) processing [25-27], and tau accumulation [21, 22], highlighting its relevance for further investigation.

CK2 is a tetrameric enzyme composed of 2 regulatory β subunits (CK2β) and 2 catalytic subunits either α (CKα) or α’ (CK2α’) [16]. CK2α and CK2α’ are highly similar in structure but display differential expression and substrate specificity[28-34]. CK2α is expressed throughout the body at relatively equal levels and has hundreds of substrates, while CK2α’ expression is more restricted to the testes and the brain and has very few validated substrates [28-34]. Another important difference between these two catalytic subunits is the embryonic lethality of CK2α knock-out mice, while complete deletion of CK2α’ does not have any major defects other than male sterility [33, 34]. Recently, studies in Huntington’s disease (HD) have shown the specific upregulation of CK2α’ in cell and mouse models of HD as well as in affected brain tissues of patients with HD [17, 18]. Importantly, HD mice lacking one allele of CK2α’ showed improved synaptic function, reduced protein aggregates, and ameliorated behavioral deficits, suggesting a key role of CK2α’ in this pathology [17, 18].

CK2 was one of the first protein kinases identified to be altered in an AD brain [35, 36], but its role in AD has been somewhat controversial due to discrepancies in the ability to detect consistent alterations in different affected tissues. Early studies using pan-CK2 antibodies (non-selective for CK2α’/CK2α subunits) showed increased levels of total CK2 in the hippocampus and frontal cortex of AD mouse models and patients with AD, especially in glial cells [37] but others have reported opposite results [35, 38, 39]. In those studies where levels of CK2 were elevated, the authors associated the increase in CK2 immunoreactivity with the activation of neuroinflammatory processes and AD pathology [37]. However, to date, genetic studies testing the role of CK2 (either CK2α’ or CK2α) in AD/ADRD models are lacking.

Despite the lack of genetic evidence for the potential differential contribution between CK2α’ and CK2α to AD pathology, several studies have proposed the role of CK2 in the phosphorylation of Tau (pTau) and tau-mediated pathology. In a kinase inhibitor screening using okadaic acid-induced pTau in Neuro-2a (N2a) cells CK2 was returned as one of the kinases whose inhibition most impacted pTau[20]. CK2 has been shown to phosphorylate the phosphatase PP2a inhibitor SET (I2PP2A/SET) increasing pTau in primary neuronal cultures treated with amyloid-β (Aβ) or overexpressing human Tau (hTau) [19]. Additionally, wild-type (WT) mice overexpressing CK2 were also shown to have increased pTau, impaired synaptic plasticity, and display cognitive deficits like those seen in tauopathies [19]. Overall, this evidence suggests a role of CK2 in tauopathies including pTau accumulation, synaptic plasticity, and cognitive deficits.

Previous studies assessing the role of CK2 in tauopathy have largely focused on the CK2 holoenzyme without differentiating between the different catalytic CK2α and CK2α’ subunits. Given the substantial difference in substrate specificity between the two catalytic subunits, it is crucial to determine whether CK2α’ and CK2α contribute differently to the onset and/or progression of tauopathies. Understanding these differences is essential for designing therapeutic strategies that are both highly effective and minimize off-target effects. The lack of commercially available inhibitors that discriminate between the different catalytic subunits offers an attractive window for designing and development of specific inhibitors to selectively inhibit CK2 catalytic subunits.

Due to previous findings specifically connecting the dysregulation of CK2α’ with various neurodegenerative diseases and reports linking this subunit to the phosphorylation of Tau in different contexts, we explored the role of CK2α’ in various tauopathy models. We confirmed a specific upregulation at the RNA and protein levels of CK2α’ in affected tissues of AD and FTD patients as well as a mouse model of tauopathy (PS19). CK2α’ upregulation was specifically observed in both neurons and microglia. Importantly, genetic depletion of CK2α’ in both cell and mouse models of tauopathy (expressing Tau-P301L or Tau-P301S mutations) resulted in decreased pTau. Furthermore, PS19 mice haploinsufficient for CK2α’ showed improved synaptic density, synapse function, and cognition. However, the effects of CK2α’ haploinsufficiency were more profoundly noted in inflammation and immune processes and in the restoration of microglia morphology, cytokine levels and phagocytic activity. Overall, our data showed a large role of the CK2α’ catalytic subunit specifically in tau pathology, largely connected to the modulation of microglia functions. These studies highlight CK2α’ as an attractive therapeutic target for the treatment of AD/ADRD.

## Methods

### Mouse Lines

For this study we used the B6;C3-Tg(Prnp-MPAT-P301S)PS19Vle/J (or PS19) [40] mouse line (Jackson #008169 maintained with purchased B6C3H breeders; obtained from Dr. Karen Ashe at University of Minnesota), and CK2α’^(+/−)^ mice, originally obtained from Dr. Seldin at Boston University (Taconic biosciences TF3062)[34] and maintained on the C57Bl/6J background for several generations. We crossed PS19 and CK2α’^(+/−)^ mice and this cross generated 4 genotypes of interest WT (PS19^0/0^;CK2α’^(+/+)^), CK2α’^(+/−)^ (PS19^0/0^;CK2α’^(+/−)^), PS19 (PS19^Tg/0^;CK2α’^(+/+)^), and PS19;CK2α’^(+/−)^ (PS19^Tg/0^;CK2α’^(+/−)^) mice. For all experiments littermate WT and CK2α’^(+/−)^ controls were used. Two time points were used in experiments; pre-symptomatic (6–7 months) and symptomatic (10–12 months). All mice were housed under standard SPF conditions. All animal care and sacrifice procedures were approved by the University of Minnesota Institutional Animal Care and Use Committee (IACUC) in compliance with the National Institutes of Health guidelines for the care and use of laboratory animals under the approved animal protocol 2307-A41243.

### Human postmortem tissue

Postmortem human frontal cortex (Brodmann’s area 46) FTD patients and controls subjects was provided by NeuroBioBank of National Institutes of Health. A total of 6 female (3 control/3FTD) and 8 male (4 control/4 FTD) samples were used, and samples were age and sex matched. Ages ranged from 60–77 with the average age of all samples being 70. More detailed information on specific samples can be found in **Supp. File 1**.

### Immunoblotting

Human and mouse protein was extracted as previously described[17]. Brain tissue was homogenized with either tissue-tearor (Biospec products, Inc) or Pellet pestle motor (Kimble Kontes) in 25 mM Tris-HCl pH 7.4, 150 mM NaCl, 1 mM EDTA, 0.1% SDS, 1% Triton X-100 and samples were incubated on ice 15–30 min and vortexed at full speed for 30 s every 5 min. Additional SDS was added to samples to bring total concentration up to 2%. Mouse protein was heated at 100°C for 5 min. Samples were centrifuged at 4°C, 13,000 g for 20 min and the supernatant was collected. Protein concentration determined using the BCA protein quantification assay (Pierce).

Immunoblotting was conducted as previously described [17]. Human and mouse protein samples were separated on 4–20% SDS Criterion TGX Stain-Free gels (BioRad) at 80 V. Proteins were transferred to a nitrocellulose membrane (BioRad 0.2 μm) in Tris–Glycine Buffer (25 nM Tris-Base, 200 mM Glycine) at 25 V for 30 min in Trans Turbo Transfer system (BioRad). The membrane was blocked with 5% non-fat dry milk in TBS containing 0.5% Tween-20 (TBST) for 1 h at room temperature. Membrane was incubated in TBST containing 2.5% milk overnight at 4°C. The next day, membranes were incubated in secondary antibody (Amersham ECL HRP Conjugated Antibodies 1:5000) in TBST containing 2.5% milk) for 1 h at room temperature. Followed by band detection using SuperSignal Chemiluminiscent substrate (Thermo Scientific) and GE ImageQuant Las4000 mulit-mode imager. Primary antibodies and their concentrations are as follows: CK2α’ (1:2000; Rabbit, Novus NB100-379) and GAPDH (1:5000; Mouse, Santa cruz sc-365062).

### Cell culture: Culturing, transfections, and immunocytochemistry

Neuro-2a cells (N2a; ATCC CCL-131) were originally obtained from Dr. Veena Prahlad (University of Iowa). Cells were cultured at 37°C 5% CO_2_ in Dulbecco’s modified Eagle’s medium (DMEM, Genesee) supplemented with 10% fetal bovine serum (FBS), 100 U ml^−1^ penicillin/streptomycin as previously described [20, 41, 42]. For silencing experiments cells were simultaneously plated in 12-well plates for RNA-extraction or 24-well plates containing Matrigel matrix (Corning) coated glass coverslips for immunocytochemistry at a density of 80,000 cells/cm^2^.

24 hours after plating all cells were transfected with siRNA for CK2α (Qiagen SI02007180, Rn_RGD:621663_4 FlexiTube siRNA), CK2α’ (Qiagen SI00961072 Mm_Csnk2a2_4 Flexitube siRNA, Qiagen SI00961051 Mm_Csnk2a2_1 FlexiTube siRNA, Qiagen SI00961058 Mm_Csnk2a2_2 FlexiTube siRNA, and Qiagen SI00961065 Mm_Csnk2a2_3 FlexiTube siRNA pooled) or AllStars negative scrRNA control (Qiagen S103650318) using DharmaFECT 1 (Horizon discovery) at a final concentration of 25 nM in cell medium. 24 hours after siRNA transfection cells were transfected with either pEGFP-C1 plasmid (Clontech) or prk5-Tau-P301L-EGFP (Addgene #46908) [43] using jetOptimus transfection reagents (Polyplus). 24 hours after transient transfection RNA was extracted as described below and cells were fixed with 4% PFA for immunocytochemistry.

For immunocytochemistry cells were blocked in 5% NGS in TBST (0.3% triton x-100 in TBS) for 1 hour, then incubated in primary antibody diluted in overnight 5% NGS in TBST (0.3% triton x-100 in TBS) (pTau (Ser202, Thr205) AT8 (1:500; Mouse Invitrogen Mn1020)). Secondary Alexa-fluorophore-conjugated antibodies (Invitrogen) were added (1:200 in TBST with 5% NGS) for 1 h at room temperature. Coverslips were mounted in ProLong Gold Antifade with DAPI (Invitrogen) and subsequently imaged.

### Immunohistochemistry

Immunohistochemistry experiments were conducted as previously described [17, 44, 45], and stained with either fluorescent secondaries or carried through 3,3'-diaminobenzidine (DAB) staining. Mice were anesthetized with Avertin (250 mg/kg Tribromoethanol) and perfused intracardially with tris-buffered saline (TBS) (25 mM Tris-base, 135 mM Nacl, 3 mM KCl, pH 7.6) supplemented with 7.5 mM heparin. Brains were dissected, fixed with 4% PFA in TBS at 4°C for 4–5 days, cryoprotected with 30% sucrose in TBS for 4–5 days and embedded in a 2:1 mixture of 30% sucrose in TBS:OCT (Tissue-Tek). Brains were cryo-sectioned into 16 μm-thick coronal slices and stored in a 50% glycerol-50% TBS (25 mM Tris-base, 125 mM NaCl, 3 mM KCl, pH 7.6) solution at −20°C. For each experiment, three hippocampal containing sections were used per mouse.

For immunofluorescent staining, sections were blocked in 5% normal goat serum (NGS) in TBST for 1 h at room temperature. Primary antibodies were incubated overnight at 4°C in TBST containing 5% NGS. Secondary Alexa-fluorophore-conjugated antibodies (Invitrogen) were added (1:200 in TBST with 5% NGS) for 1 h at room temperature. Slides were mounted in ProLong Gold Antifade with DAPI (Invitrogen) and subsequently imaged. Primary antibodies used and dilutions are as follows: GFAP (1:2000; Chicken Millipore Sigma AB5541), Iba1 (1:500; Rabbit Fujifilm Wako 019-19741), Iba1 (1:500, Goat Fujifilm Wako 011-27991), NeuN (1:1000; Mouse Millipore MAB377), PSD95 (1:500; Rabbit Invitrogen 51-6900) and Vglut1 (1:500; Guinea pig Millipore AB5905). Imaging of immunofluorescent slices was conducted using a confocal microscope (Stellaris, Lecia). For NeuN, Iba1, and GFAP imaging 20x tiles were acquired with a 16um z-dimension and 1um step size. For PSD95 and VGlut1 images were acquired as described for synapse analysis.

For DAB staining antigen retrieval was performed using either Tris-EDTA pH = 9.0 (Biolegend 422703) or Rodent decloaker (Biocare medical RD913) at 80°C for 30 minutes. Sections were blocked at room temperature in TBST containing 10% NGS for 1 hour, then incubated in primary antibodies overnight at 4C in TBST containing 5% NGS. Sections were incubated in biotin-conjugated secondaries (Jackson Immuno Research Labs Biotin-SP (long spacer) AffiniPure) in TBST containing 5% NGS for 1 hour, then blocked in 3% hydrogen peroxide for 20 minutes. Sections were incubated in tertiary antibodies (VECTASTAIN Elite ABC, HRPS kit, Vector laboratories) in TBST containing 5% NGS for 1 hour per manufacturer instructions. Sections were then incubated in DAB chromogen diluted in DAB substrate buffer (Biolegend) for sufficient staining. For counterstaining sections were incubated in 0.1% Cresyl violet for 10–12 minutes at 37°C. Slides were then dehydrated and cleared in a series of ethanol and xylene washes before being mounted in Permount mounting medium (Fisher scientific). Primary antibodies used and dilutions are as follows: pTau (Ser202, Thr205) AT8 (1:1000; Mouse, Invitrogen Mn1020) and CD68 [RM1031] (1:2500; Rabbit, Abcam ab303565). DAB images were taken at 10x on a hybrid microscope using the brightfield settings (Echo Revolve) and at 40x on the EasyScan One slide scanner (Motic).

### in situ hybridization

Tissues were collected as previously described in the immunohistochemistry procedure. Slices were mounted on superfrost plus glass slices using PBS and stored at −80°C overnight before *in situ* hybridization. RNAscope was performed according to the ACDbio manufacturer’s protocol using a custom probe for *Csnk2a2*. Immediately following RNAScope protocol, the slides were blocked with 10% Normal Goat Serum (NGS) in Co-detection antibody diluent (CDD) for 1 hour. Anti-IBA1 (Rabbit, Fujifilm Wako 019-19741) was diluted at 1:200 in 5% NGS with CDD and slides were incubated overnight. The next day secondary Alexa-fluorophore-conjugated antibodies (Invitrogen) were added (1:40 in CDD with 5% NGS) for 2h at room temperature. Slides were mounted in ProLong Gold Antifade with DAPI (Invitrogen) and subsequently imaged.

### Tau pathology type

Tau pathology type analysis was conducted by 3 independent and blinded investigators. Tau pathologies were defined based on previous publications [46-49] and based on the pattern of AT8 + staining. Briefly as described by Shi et al 2017 [46] type 1; displays mossy fiber and sparse and diffuse cell body staining in the dentate gyrus (DG), type 2; displays compact dense tangle like cell body staining in the DG and CA3 with some sparse CA1 cell body staining, type 3; staining is seen in the neuropil of the stratum radiatum of the CA1 staining along with staining of dendrites from pyramidal neurons and sparse cell body staining, type 4; dense fragmented, dotted, and grainy staining is observed all over the hippocampus. The percentage of pathology for the cohort for each type was determined by the following calculation; ((number of mice with pathology type)∕(total number of mice in cohort))×100.

### Image quantification

All image quantifications were conducted using either QuPath [50] or Image J (FIJI) [51] and were blinded to the experimenter. For all quantifications images names were either blinded in FIJI using the BlindAnalysis plugin or in Qupath using the automated image masking tool. Quantifications were performed with at least two blinded experimenters.

Quantification of NeuN was done automatically using QuPath cell detection. Identical ROIs were applied to each image for all 3 regions of the hippocampus. The cell detection tool was used to automatically detect NeuN + cells based on identical thresholding settings applied to all images. Quantification of Iba1 + and GFAP + cell counts was conducted as we previously described [44, 45]. An average projection of 20x tiled z-stack confocal image was generated in FIJI and used for counting. Two consistent ROIs (~ 3900 um^2^) were drawn within the CA1, CA3, and DG for counts and the cell counter FIJI plugin was used to count cells manually. A cell was determined to be Iba1 + or GFAP + based on the presence of DAPI within the stained area. Results were averaged and normalized per mm^2^ and reported as animal averages (2 ROIs/slice; 3 slices/animal). Quantification of DAB positive percent area was quantified using either the Colordevonvolution2 plugin in FIJI [52] or in QuPath. Briefly a threshold of positive DAB staining was determined and applied to all images to then calculate the percentage positive area.

Quantification of PSD95 + puncta within Iba1 + microglia were performed using Imaris software (Bitplane). A confocal microscope (Stellaris, Lecia) was used to acquire images in the CA1 stratum radiatum region of the hippocampus with a 40x objective and 3x zoom factor with 0.34 size z-steps for a total of 15 steps. Lecia Stellaris software lighting processing was applied before analysis. Images were then 3D rendered in Imaris and cropped to isolate individual Iba1 + cells (2 per slice). Iba1 + surfaces were generated and used to mask PSD95 + signal. The masked PSD95 + signal was then used to create spots, to ensure it was located within Iba1 + signal. The number of spots within the Iba1 + surfaces per cell soma defined as the region where Iba1 + signal colocalized with DAPI and within the whole Iba1 + cell surface were counted and reported as a number.

### Synapse analysis

Synapse analysis was conducted as previously described [17, 45, 53-55]. Immunohistochemical staining was conducted as described above with Vglut1 and PSD95, with blocking increased to 20% NGS + TBST for 2 hours and primary and secondary antibodies diluted in 10% NGS + TBST. Fluorescent images from the molecular layer of the CA1 were taken on a confocal microscope (Stellaris, Lecia) at 63x with z-step dimension of 0.34 μm with 15 steps were generated. Lecia stellaris software lightning processing was applied to images post-acquisition and edited images were used for analysis. Maximum projections of three sections per slice were generated. Puncta analyses were conducted blinded using the PunctaAnalyzer Plugin (Durham,NC,USA) in FIJI as previously described [17, 45, 53-55]. Data is represented as slice averages with three slices per animal.

### Electrophysiology

Mice were anesthetized with isoflurane (2%) and decapitated for slice preparation. After decapitation, the whole brain, containing the two hippocampi, was removed into ice-cold solution consisting of: sucrose 189 mM, glucose 10 mM, NaHCO3 26 mM, KCl 3 mM, MgSO4 5 mM, CaCl2 0.1 mM, NaH2PO4 1.25 mM. Brains were positioned on the stage of a vibratome slicer and cut to obtain transverse hippocampal slices (350 μm thick; LEICA VT1000S). Slices were incubated at room temperature in artificial cerebrospinal fluid (aCSF) continuously oxygenated for 45 min-1 h before use. aCSF contained NaCl 124 mM, KCl 5 mM, NaH2PO4 1.25 mM, MgSO4 2 mM, NaHCO3 26 mM, CaCl2 2 mM, glucose 10 mM; gassed with 95% O2, 5% CO2. The pH was adjusted to 7.4 with NaOH. All experiments were carried out at 30–34°C, during which the slices were continuously perfused with solution.

Field excitatory postsynaptic potentials (fEPSPs) were recorded in the CA1 region of the hippocampus. fEPSPs in the CA1 region of the hippocampus were evoked with a stimulating electrode placed on the Schaffer collateral (0.33 Hz) using brief current pulses (200 μs, 0.1–0.2 mA). Extracellular recording electrodes were filled with aCSF. Stimulation was adjusted to obtain a fEPSP peak amplitude of approximately 1 mV during control conditions. After a stable fEPSP baseline period of 10 min, LTP was induced by a Theta-burst stimulation (TBS) consisting of a series of 10 bursts of 5 stimuli (100 Hz within the burst, 200 ms interburst interval), which was repeated 4 times (5 s apart). Data were filtered at 3 kHz and acquired at 10 kHz using pCLAMP 10.2 software (Molecular Devices, RRID: SCR_011323).

A stimulus-response curve (0.05–0.4 mV, mean of five fEPSPs at each stimulation strength) was compiled for the different mice used. For paired-pulse ratio experiments, two fEPSPs were evoked 40 ms apart for 0.5 min at baseline frequency (6 times) at the beginning of the baseline recording. The PPR was expressed as the amplitude of the second fEPSP divided by the amplitude of the first fEPSP.

### Behavior

Behavioral testing was performed with the support and guidance of the Mouse Behavior Core at University of Minnesota. Mice were handled daily for a minimum of one week prior to all behavioral testing. Mice were habituated to behavior room for a minimum of 30 minutes each day prior to beginning of task. All tasks were recorded and tracked using ANYmaze software (Stoelting Co., Wood Dale, Illinois) and overhead cameras.

Barnes maze was conducted on a 20 hole Barnes Maze (San Diego Instruments) divided into 4 quadrants and a center zone under 450-500lux light intensity, with black and white visual cues placed around the room, as similarly described [56-58]. Mice were placed in the center under a covering with lights off, lights were turned on and covering lifted within 30 seconds. The training phase consisted of four 3-minute trials per mouse for 5 days, with an inter trial interval of approximately 20–30 minutes. The maze was thoroughly cleaned with 70% between all mice and rotated between trials to avoid olfactory cues. Mice were allowed to explore the maze and primary latency, or the time to first explore the escape hole, was recorded. The trial either concluded when the mouse entered the escape hole, or at 3 minutes at which point the mouse was gently guided to the escape box. For the probe day the escape hole was covered with an identical covering used on all other holes, mouse was placed on the maze in the same manner as training. The time spent in each maze zone was then recorded. Spatial strategy was determined by a blinded investigator.

Open field was conducted in 40cmx40cm boxes with a 150-200lux overhead light. Mice were placed in boxes and behavior was recorded for 1 hour. Boxes were thoroughly cleaned between mice with 70% ethanol.

### RNA extraction and qPCR

RNA was extracted from cells and mouse striatal tissues by using the RNeasy extraction kit (Qiagen) according to the manufacturer’s instructions. cDNA was prepared using the Superscript First Strand Synthesis System (Invitrogen). SYBR green based PCR was performed with SYBR mix (Roche). The qPCR amplification was performed using the LightCycler 480 System (Roche). Each sample was tested in triplicate and normalized to GAPDH levels.

For qPCR the following primers were used: CK2α’ **FWD-**CGACTGATTGATTGGGGTCT **REV-** AGAATGGCTCCTTTCGGAAT; CK2α **FWD-**TCCCCATGCTGTGACAATAA **REV-**AAGACCCTGTGTCACGAACC; CK2β **FWD-** AGTCCTCCAGACACCACCAC **REV-**GACTGGGCTCTTGAAGTTGC; huTau **FWD-** GCTGGCCTGAAAGCTGAAGA **REV-** CGTTTTACCATCAGCCCCCT.

### RNA-Seq Analysis

RNA was extracted as previously described and RNA-Sequencing was conducted by Novogene (Sacramento, CA). Gene expression analysis was carried out using the CHURP pipeline (https://doi-org.ezp2.lib.umn.edu/10.1145/3332186.3333156) using n = 4–7 mice/genotype with female(F)/male(M) ratios as follows; 4 WT (2F/2M), 5 CK2α’^(+/−)^ (2F/3M), PS19 (3F/4M) (3 high pathology and 4 low pathology), and 4 PS19;CK2α’^(+/−)^ (3F/1M). Differential gene expression was determined with DESeq2 using default setting [59] (v1.46.0). Genes with a FDR < = 0.05 were considered significant. Outliers’ identification was performed using Cook’s distance (DESeq2). Driver factors of gene expression variance (genotype and/or sex) were evaluated using R (v4.4.2) package variancePartition (1.36.3). Pathway and clustering analysis were completed gProfiler2 [60] (v0.2.3) and clusterProfiler (v4.14.6). Data visualization was done using various R graphic packages, including ggplot2, ggraph, and DESeq2 visualization functions. The RNA-seq data set generated in this manuscript has been deposited at GEO (accession number GSE298505). The reviewer token to access the GEO deposited data is **mlwxmuactlibryl**.

### Microglia morphology

Microglia morphology measurements were adopted from previously described protocols [61]. Slices were stained with Iba1 + as described in the immunohistochemistry methods. Nine total cells were imaged per animal across 3 slices with 3 cells per slice. Images were taken on a confocal microscope (Stellaris, Lecia) at 40x magnification with a zoom factor of 2.5. Z-stacks were acquired with a Z-step size of 0.3 μm, covering the entire slice. Maximum projection of z-stacks were generated via FIJI for analysis. For image analysis, brightness and contrast were adjusted to subtract background noise before converting the image into a binary black-and-white version of the original. The paintbrush and despeckle functions were used to remove any additional noise before applying the skeletonizing function. Finally, the AnalyzeSkeleton plugin [62] was used to quantify the resulting skeleton image.

### Cytokine proteome profiling

The Proteome Profiler Mouse Cytokine Array Panel (ARY006, R&D Systems) was used to detect the levels of cytokine/chemokine as per manufacturer’s instructions. Frozen hippocampi from 9 months old PS19 and PS19;CK2α’^(+/−)^ mice were homogenized in PBS containing Halt protease inhibitor cocktail and phosphatase inhibitors (Fisher Scientific) and 1% triton X-100 (Sigma). Samples were stored at −80°C for 15 min, thawed and centrifuged at 10,000 × g for 5 min to remove cell debris. 300 μg of protein was applied to each membrane and the manufacturer’s protocol was followed. A total of n = 4 mice/genotype with a female(F)/male(M) ratio (2F/2M PS19 and 2F/2M PS19;CK2α’^(+/−)^) were analyzed. Imaging was conducted as described for immunoblotting. Spot intensity was quantified using FIJI software and a batch correction was applied to normalize data. Membrane spots corresponding to 40 different cytokines or chemokines were measured as outlined in manufacturer protocol.

### Statistical analyses and data representation

For electrophysiology experiments data were analyzed using Clampfit 10.2 software (pCLAMP, Molecular Devices, RRID: SCR_011323). Data are presented as mean ± SEM. To estimate changes in synaptic efficacy, STP was quantified by comparing the mean fEPSP amplitude during 1 minute after the application of the TBS. LTP was quantified as for STP but 40 minutes after the protocol. Graphs were obtained using SigmaPlot 14.0. Before applying any statistical comparison, the data were subjected to Shapiro- Wilk normality and equal variance tests. For any comparisons between two groups, two-paired Student’s t-test was used. For multiple comparisons to the same control, One-way ANOVA and Holm-Sidak test was used. P-values less than 0.05 were considered statistically significant.

For all other experiments GraphPad Prism (GraphPad, San Diego, CA, USA) was used to create graphs and conduct statical testing. For all other experiments data is represented as mean ± SEM. Statistical analyses were performed using paired Student’s t-test, unpaired student’s t-test, one-way or two-way ANOVA with post-hoc analysis, as indicated in each figure legend. A p-value < 0.05 was considered significant.

## Results

### Levels of Protein kinase CK2α’ are increased in the brains of dementia patients and mouse models of tauopathy

**Figure 1:**
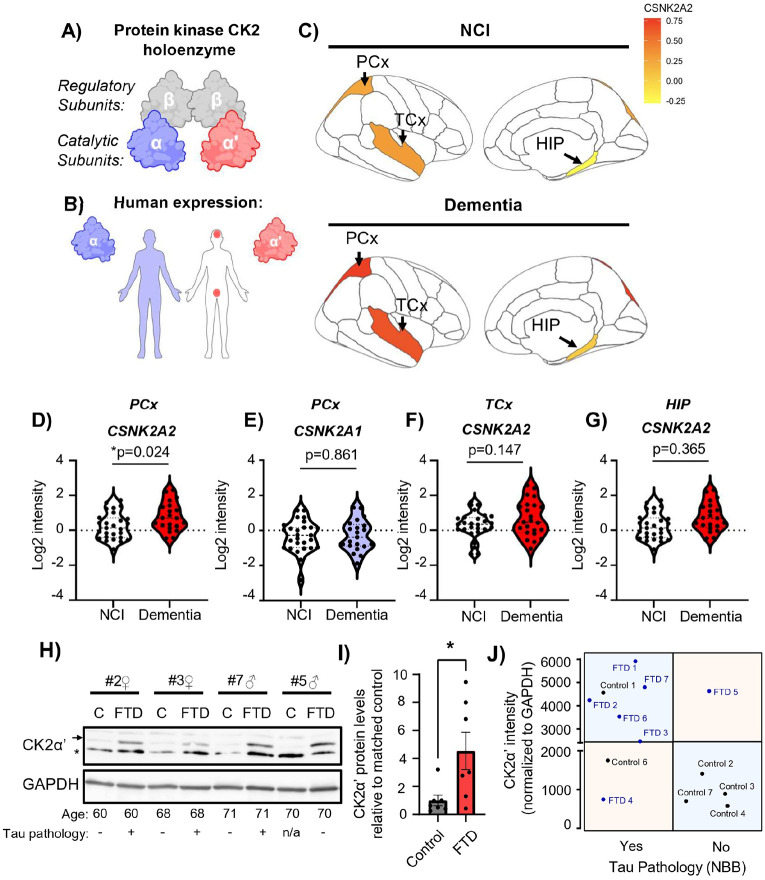
CK2α’ is increased in tissues of patients with AD/ADRD. **(A, B)** Representative diagram of CK2 holoenzyme, including regulatory and catalytic subunits and differential distribution of expression of catalytic subunits of CK2α and CK2α’ in the human body. A and B were created in BioRender (White, A. (2025) eqznm6m https://BioRender.com/). **(C)**Averaged expression of CSNK2A2 in non-cognitively impaired (NCI) and dementia samples from the Allen Brain Institute: Aging, Dementia and TBI study; parietal cortex (PCx), Temporal cortex (TCx), and Hippocampus (HIP) are shown (patients with TBI were excluded from the analyses). **(D-G)** Quantification of the expression of CSNK2A2 and CSNK2A1 in PCx (**D, E**), TCx (**F**), and HIP (**G**) from NCI and dementia from the Allen Brain Institute: Aging, Dementia and TBI study (n=20-27/group). Significance determined by unpaired t-test, *p<0.05. **(H)** Immunoblot for CK2α’ in protein lysates from frontal cortex of age and sex matched healthy controls (C) and patients with FTD. Arrow indicates CK2α’ specific band, asterisk indicates unspecific band. **(I)** CK2α’ protein levels were normalized to GAPDH and shown relative to age/sex matched control, quantified using ImageJ software (n=7/group). Statistical analyses were conducted using unpaired t-test with welch’s correction p=0.0377. Data are shown as mean ± SEM. (**J**) Relationship between CK2α’ levels from (I) and the presence of Tau pathology reported by the NIH biobank for those samples. Horizontal line represents the group CK2α′ intensity average normalized by GAPDH.

There are two different genes (*Csnk2a1* and *Csnk2a2*) that code for two different CK2 catalytic subunits CK2α and CK2α’, respectively. These two catalytic subunits share a high percent of homology and they can arrange in various combinations (α-α, α-α’ or α’-α’) depending on their expression and tissue availability although the canonical arrangement in the brain is expected to be α-α’ (Fig. 1A, B) [16, 28-34]. To determine whether CK2 catalytic subunits are altered in patients with dementia we utilized the RNA-seq data from the Allen Brain Institute: Aging, Dementia, and Traumatic Brain Injury (TBI) Study. In this dataset, three different regions were available: parietal cortex (PCx), temporal cortex (TCx) and hippocampus (HIP). Analyzing this dataset [63] we found a specific increase in the expression of *CSNK2a2*, encoding for CK2α’, but not in *CSNK2a1*, encoding for CK2α, in patients with dementia compared to non-dementia controls in the PCx (Fig. 1C-E). We also found a trend towards increased CK2α’ in the TCx and HIP although data did not reach statistical significance (Fig. 1C, F, G). Importantly, we also observed a significant increase in CK2α’ protein levels in the frontal cortex of FTD patients compared to age and sex-match controls (Fig. 1H, I, **Supp. File 1, Supp. File 2**). Enhanced levels of CK2α’ also associated with the presence of tau pathology previously assessed in those samples using the AT8 antibody (Fig. 1J, **Supp. File 1**). The majority of FTD samples grouped in the top left quadrant representing high levels of CK2α’ and the presence of tau pathology, while the majority of control samples grouped in the bottom right quadrant representing low levels of CK2α’ and absence of tau pathology. For those two control samples (#1 and #6) in which tau pathology was observed, the AT8 phospho-tau (pTau) signal and distribution differed from the canonical tau pathology associated with dementia and individuals lacked classical cognitive decline (**Supp. File 1**).

**Figure 2:**
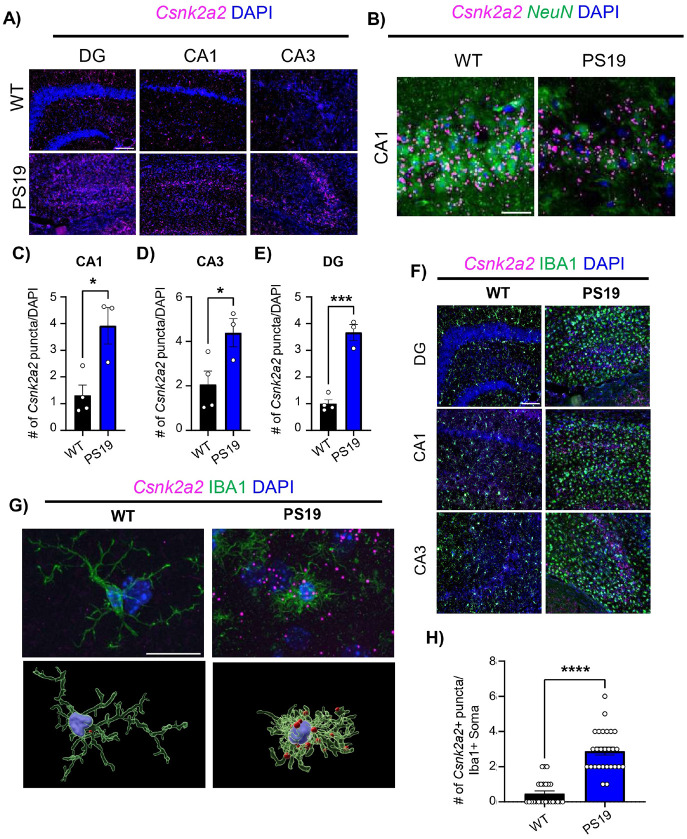
Csnk2a2 expression is increased in both neurons and microglia in the hippocampus of PS19 mice. **(A)** Csnk2a2 in situ hybridization images from symptomatic WT and PS19 hippocampus. Left panels show 10x images of hippocampal cell layers with Csnk2a2 RNA probe and DAPI, scale bar=50 μm. White boxes indicate zoomed inlets shown on the right panels at 20x, scale bar=10 μm. **(B)** Csnk2a2 in situ hybridization and NeuN immuno-fluorescence images from symptomatic WT and PS19 CA1. Scale bar=20 μm. (**C-E**) Quantification of Csnk2a2 puncta in the CA1 (p=0.0163) (**C**), CA3 (p=0.0466) (**D**) and DG (p=0.0003) (**E**) region quantified within the cell layer identified by DAPI, (n=3-4 mice/genotype). **(F)** Representative images Csnk2a2 RNA probe and Iba1 immunofluorescence in symptomatic WT and PS19 hippocampus. **(G)** Top: representative image of single Iba1+ cell in the Stratum Radiatum (CA1) with Csnk2a2 RNA Scope. Scale bar is 20 μm. Bottom: 3D rendering of cells Iba1+ cells containing Csnk2a2 puncta showing puncta reside within the cell. **(H)** Quantification of the number of Csnk2a2+ puncta/Iba1+ soma (p<0.0001,n=3-4 mice/group, 8-9 cells/mouse, every data point is a cell). All Data shown are mean ± SEM. Statistical analyses were conducted using unpaired t-test * p<0.05, *** p<0.001, **** p<0.0001.

To examine whether CK2α’ was also elevated in mouse models of tauopathy we utilized the PS19 mouse model, which expresses human tau with a P301S mutation associated with familial forms of FTD and other tauopathies [40]. Pathology and neuronal loss in PS19 mice are seen largely in the hippocampus, although it does spread to other brain regions including the neocortex, entorhinal cortex and amygdala [40, 64]. We conducted RNA in situ hybridization for CK2α’ in the hippocampus of PS19 mice at a symptomatic age (10–12 months), defined as the age at which animals present overt tau pathology and cognitive decline [40, 65, 66]. We observed a significant increase in CK2α’ in all regions of the hippocampus specially in the pyramidal and granular layers, that colocalized with NeuN staining, pointing to enhanced expression in neurons (Fig. 2A-E). Expression outside the neuronal layer was also observed which associated with enhanced CK2α’ levels in the microglia (Iba1+) of PS19 mice compared to WT (Fig. 2F-H). Single cell RNA-Seq studies available in The Alzheimer’s Cell Atlas (TACA) [67-71] confirmed increased CSNK2a2 expression in patients with AD in both microglia and neurons in the occipital cortex (OL) and entorhinal cortex (EC) respectively (**Supp. Figure 1**). However, studies in prefrontal cortex (PFC) and superior frontal gyrus (SFG) within the frontal lobe revealed enhanced expression of CSNK2a2 in cells other than neurons and microglia, suggesting a cell-specific upregulation of CSNK2a2 that seems to be brain region-dependent. Overall, these results demonstrate a specific upregulation of CK2α’ in brain affected regions of patients with dementia and in hippocampal neurons and microglia of mouse models of tauopathy.

### Genetic depletion of Protein kinase CK2α’ impacts tau pathology in vitro and in vivo

**Figure 3:**
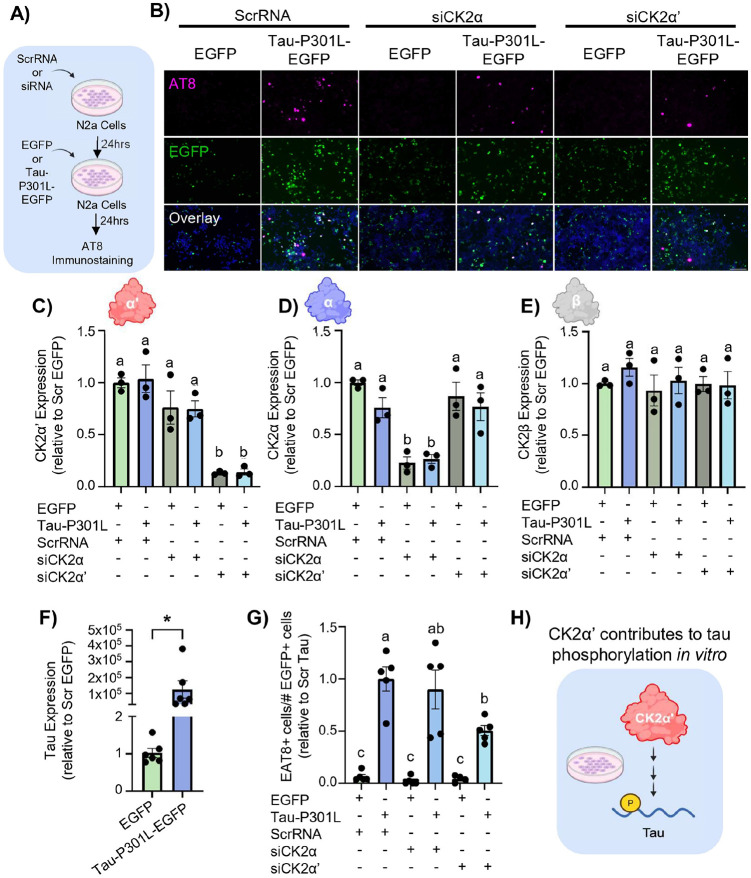
Silencing CK2α’ reduces pTau levels in N2a cells expressing Tau-P301L. **(A)** Schematic of experimental design for N2a cell transfection and immunostaining. **(B)** Representative images of AT8 immunostaining and EGFP fluorescence in N2a cells transfected with either EGFP or Tau-P301L-EGFP and treated with ScrRNA, siCK2α, or siCK2α’. Scale bar= 100 μm. **(C-E)** RT-qPCR analysis of mRNA expression levels of CK2α’ (Scr-EGFP vs. siCK2α'-EGFP p=0.0003, Scr-EGFP vs. siCK2α'-Tau-P301L p=0.0003, Scr-Tau-P301L vs. siCK2α'-EGFP p=0.0002, Scr-Tau-P301L vs. siCK2α'-Tau-P301L p=0.0002, siCK2α-EGFP vs. siCK2α'-EGFP p=0.0049, siCK2α-EGFP vs. siCK2α'-Tau-P301L p=0.0056, siCk2α-Tau-P301L vs. siCK2α'-EGFP p=0.0059, siCk2α-Tau-P301L vs. siCK2α'-Tau-P301L p=0.0067) **(C)**, CK2α (Scr-EGFP vs. siCK2α-EGFP p=0.0008, Scr-EGFP vs. siCk2α-Tau-P301L p=0.0013, Scr-Tau-P301L vs. siCK2α-EGFP p=0.0161, Scr Tau-P301L vs. siCk2α Tau-P301L p=0.0262, siCK2α EGFP vs. siCK2α' EGFP p=0.004, siCK2α-EGFP vs. siCK2α'-Tau-P301L p=0.0144, siCk2α-Tau-P301L vs. siCK2α'-EGFP p=0.0065, siCk2α-Tau-P301L vs. siCK2α'-Tau-P301L p=0.0235) **(D)**, and CK2β **(E)** in transfected N2a cells. Expression levels were normalized to GAPDH and presented relative to ScrRNA-EGFP condition (n=3/condition). **(F)** RT-qPCR analysis of mRNA expression levels of huTau expression in N2a cells transfected with EGFP or Tau-P301L-EGFP transfected N2a cells. Expression levels were normalized to GAPDH and presented relative to EGFP condition (p=0.0464, n=6/condition). **(G)** Quantification of AT8+ cells normalized to the number of EGFP+ cells, presented relative to ScrRNA-Tau-P301L condition. (n=4/condition; Scr-EGFP vs. Scr-Tau-P301L p<0.0001, Scr-EGFP vs. siCk2α-Tau-P301L p<0.0001, Scr-EGFP vs. siCK2α'-Tau-P301L p=0.0334, Scr-Tau-P301L vs. siCK2α-EGFP p<0.0001, Scr-Tau-P301L vs. siCK2α'-EGFP p<0.0001, Scr-Tau-P301L vs. siCK2α'-Tau-P301L p=0.013, siCK2α-EGFP vs. siCk2α-Tau-P301L p<0.0001, siCK2α-EGFP vs. siCK2α'-Tau-P301L p=0.0163, siCk2α-Tau-P301L vs. siCK2α'-EGFP p<0.0001, siCK2α'-EGFP vs. siCK2α-Tau-P301L p=0.0357). All data shown as mean ± SEM. Statistical analyses were conducted using one-way ANOVA with tukey’s multiple and displayed with compact letter display (**C,D,E,G**) or unpaired t-test *p<0.05 (**F**). **(H)** Diagram summarizing results of experiment in B. **A** and **H**were created in BioRender (White, A. (2025) https://BioRender.com/eqznm6m).

Previous studies have shown that CK2 holoenzyme regulates tau phosphorylation in various cell and mouse models of AD [19, 72], but whether CK2α’ subunit specifically contributes to this phenomenon is unknown. To explore whether CK2α’ has any role in the regulation of tau phosphorylation and pathology we first explored the impact of silencing CK2α or CK2α’ in Neuro2a (N2a) cells expressing a pathological tau variant (Tau-P301L). N2a cells were treated with either scrambled RNA (ScrRNA) or silencing RNA for CK2α (siCK2α) or CK2α’ (siCK2α’), and transfected with either Tau-P301L-EGFP [43] or EGFP empty vector (control) (Fig. 3A). The AT8 phospho-tau (pTau) antibody was then used to examine the impact of silencing CK2α and CK2α’ on tau pathology (Fig. 3B). First, we confirmed the efficacy of the siRNAs decreasing the expression of CK2α or CK2α’ without altering the other subunits (Fig. 3C-E) and the increased expression of Tau in N2A transfected cells (Fig. 3F). As expected, the levels of AT8 increased upon expression of Tau-P301L in N2A control cells (Fig. 3B, G). Importantly, AT8 levels decreased in Tau-P301L transfected cells treated with siCK2α’ (p = 0.0130) but not siCK2α relative to Tau-P301L ScrRNA condition (p = 0.9722) (Fig. 3B, G). Our data demonstrates an impact of the CK2α’ subunit specifically on pTau accumulation *in vitro* (Fig. 3H).

**Figure 4:**
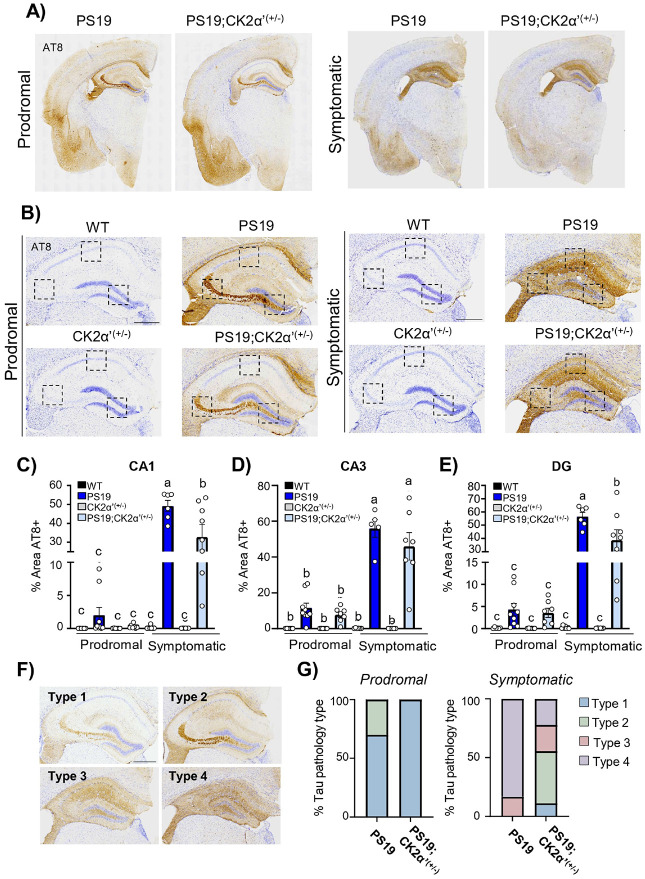
CK2α’ haploinsufficiency reduced tau pathology in PS19 mice. **(A)** Representative whole-slice sections immunostained for pTau (AT8;Ser202/Thr205) in prodromal and symptomatic mice, scale bar= 1000 μm. **(B)** Higher magnification images of hippocampus (CA1, CA3, and DG) from sections shown in (A). Scale bar=500 μm. **(C-E)** Quantification of AT8+ immunoreactivity (% area) in the CA1, CA3 and DG regions in prodromal and symptomatic cohorts (n=6-9 mice/genotype). Data represented as mean ± SEM. Statistical analyses were conducted using two-way ANOVA with tukey’s posthoc analysis and displayed with compact letter display. (**CA1 significant comparisons**: prodromal: WT vs. symptomatic: PS19 p<0.0001, prodromal: WT vs. symptomatic: PS19;CK2α’^(+/−)^ p<0.0001, prodromal: PS19 vs. symptomatic: PS19 p<0.0001, prodromal: PS19 vs. symptomatic: PS19;CK2α’^(+/−)^ p<0.0001, prodromal: CK2α’^(+/−)^ vs. symptomatic: PS19 p<0.0001, prodromal: CK2α’^(+/−)^ vs. symptomatic: PS19;CK2α’^(+/−)^ p<0.0001, prodromal: PS19;CK2α’^(+/−)^ vs. symptomatic: PS19 p<0.0001, prodromal: PS19;CK2α’^(+/−)^ vs. symptomatic: PS19;CK2α’^(+/−)^ p<0.0001, symptomatic: WT vs. symptomatic: PS19 p<0.0001 , symptomatic: WT vs. symptomatic: PS19;CK2α’^(+/−)^ p<0.0001, symptomatic: PS19 vs. symptomatic: CK2α’^(+/−)^ p<0.0001, symptomatic: PS19 vs. symptomatic: PS19;CK2α’^(+/−)^ p=0.0075, symptomatic: CK2α’^(+/−)^ vs. symptomatic: PS19;CK2α’^(+/−)^ p<0.0001; **CA3 significant comparisons:** all significant comparisons p<0.0001; **DG significant comparisons:** prodromal: WT vs. symptomatic: PS19 p<0.0001, prodromal: WT vs. symptomatic: PS19;CK2α’^(+/−^p<0.0001, prodromal: PS19 vs. symptomatic: PS19 p= <0.0001, prodromal: PS19 vs. symptomatic: PS19;CK2α’^(+/−)^ p<0.0001, prodromal: CK2α’^(+/−)^ vs. symptomatic: PS19 p<0.0001, prodromal: CK2α’^(+/−)^ vs. symptomatic:PS19;CK2α’^(+/−)^ p<0.0001, prodromal: PS19;CK2α’^(+/−)^ vs. symptomatic: PS19 p<0.0001, prodromal :PS19 CK2α’^(+/−)^ vs. symptomatic: PS19;CK2α’^(+/−)^ p<0.0001, symptomatic: WT vs. symptomatic: PS19 p<0.0001, symptomatic: WT vs. symptomatic: PS19;CK2α’^(+/−)^ p<0.0001, symptomatic: PS19 vs. symptomatic: CK2α’^(+/−)^ p<0.0001, symptomatic: PS19 vs. symptomatic :PS19;CK2α’^(+/−)^ p=0.0186, symptomatic: CK2α’^(+/−)^ vs. symptomatic:PS19;CK2α’^(+/−)^ p<0.0001) **(E)** Representative pTau pathology types. **(F)** Proportion of each pTau pathology type in prodromal and symptomatic cohorts (n=7-10 mice/genotype). Fisher’s exact t-test 7m p=0.11 & 12m p=0.0587.

We then generated a PS19 mouse haploinsufficient for CK2α’ (PS19;CK2α’^(+/−)^)(**Supp. Figure 2A-F**), to assess the impact of depleting CK2α’ on survival, tau pathology and symptomatology *in vivo*. We then assessed tau phosphorylation in the hippocampus and overlaying cortex of mice at a prodromal stage (~ 7 moths) and at a late symptomatic age (~ 10–12 moths)[40] (Fig. 4A-B, **Supp. Figure 3A-B**). In the prodromal age no significant differences were observed in the accumulation of AT8 in PS19 and PS19;CK2α’^(+/−)^ in all three examined regions of the hippocampus (CA1, CA3, and DG) and overlaying cortex (Fig. 4C-E, **Supp Fig. 3C**). As expected, in the late symptomatic groups we observed a significant increase in AT8 + area in both the PS19 and PS19;CK2α’^(+/−)^ mice in the CA1, CA3, and DG and cortex relative to all other groups (Fig. 4C-E, **Supp. Figure 3C**). However, PS19;CK2α’^(+/−)^ mice showed a significant decrease in AT8 signal compared to PS19 in the CA1, DG, and cortex (CA1; p = 0.0075, DG; p = 0.0186 and Cortex; p < 0.0001) (Fig. 4C, E, **Supp. Figure 3C**) suggesting an overall decrease in pTau upon depletion of CK2α’.

Tau pathology has been previously reported to follow a characteristic progressive pattern of deposition in the hippocampus that can be categorized into 4 pathology types [46-49] (Fig. 4F). These pathology types have been shown to correlate with hippocampal volume [46, 49] and offer a more holistic pathological assessment. We observed differential distributions of patterns between PS19 and PS19;CK2α’^(+/−)^ mice with a more dramatic alteration in late symptomatic stages where nearly all PS19 mice presented a type 4 pathology in contrast to the PS19;CK2α’^(+/−)^ that presented a variation in types with almost 50% of mice presenting type 2 pathology (prodromal p = 0.11, and symptomatic p = 0.0587) (Fig. 4G).

Interestingly, although CK2α’ seemed to play a clear role in modifying the progression of tau pathology in PS19 mice, no significant differences in survival were observed between PS19 and PS19;CK2α’(+/−) animals, with both groups showing approximately 50% mortality by 50 weeks of age (**Supp. Figure 2G**). Survival is a relatively coarse endpoint, influenced by the dysfunction of multiple neural and systemic pathways like the spinal cord and brain stem [40, 73]. While CK2α’ depletion clearly impacted tau phosphorylation and tau burden in the hippocampus and cortex, these effects were insufficient to alter the overall lifespan of this aggressive model. Nonetheless, the observed modulation of tau pathology underscored a critical role for CK2α’ in specific aspects of tau-mediated neurodegeneration. We therefore decided to further explore the molecular and cellular changes influenced by CK2α’ modulation in PS19 mice.

### CK2α’ happloinsufficiency improved hippocampal synaptic density and synaptic function in PS19 mice

**Figure 5:**
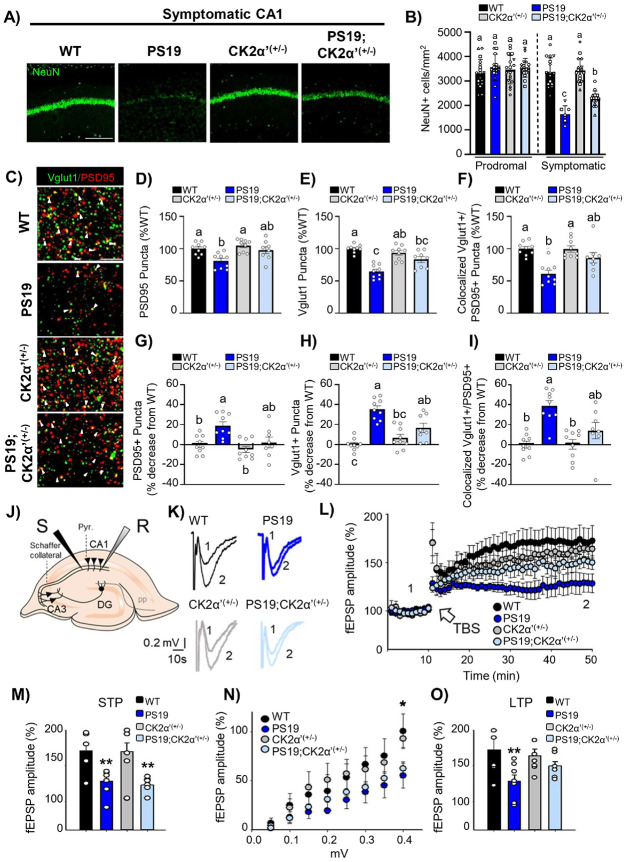
CK2α’ haploinsufficiency partially rescued hippocampal synapse loss and improved LTP PS19 mice. **(A)** Representative NeuN staining in the CA1 of symptomatic mice. Scale bar=200 μm. **(B)** Quantification of NeuN+ cells (cells/mm^2^) in the CA1 of prodromal and symptomatic cohorts (n= 3-6 mice/genotype; 2-3 slices/mouse; 1 data point= 1 slice, slices from same animal share a shape). Comparisons shown relative to age group (Significant comparisons: *Symptomatic:* WT vs. PS19 p<0.0001, WT vs. PS19;CK2α'^(+/−)^ p=0.0002, PS19 vs. CK2α'^(+/−)^ p<0.0001, CK2α'^(+/−)^ vs. PS19;CK2α'^(+/−)^ p=0.0001). **(C)** Vglut1 and PSD95 immunostaining in the CA1 of symptomatic mice. White arrow heads indicate colocalization. Scale bar= 2.5 μm. **(D-F)** Quantification of PSD95 puncta (WT vs. PS19 p=0.0445, PS19 vs. CK2α'^(+/−)^ p=0.0008) (**D**), Vglut1 puncta (WT vs. PS19 p=0.0003, WT vs. PS19;CK2α'^(+/−)^ p=0.0212, PS19 vs. CK2α'^(+/−)^ p<0.0001) (**E**) and Vglut1/PSD95 colocalized puncta (WT vs. PS19 p=0.0017, PS19 vs. CK2α'^(+/−)^ p=0.0004) (**F**) relative to WT. (n=3 mice/genotype (3 slices/mouse, 3 images/slice, 1 data point=slice average). **(G-I)** Analyses from (D-F) represented as a % decrease from WT (G: WT vs. PS19 p=0.0445, PS19 vs. CK2α'^(+/−)^ p=0.0008 H: WT vs. PS19 p=0.0003, WT vs. PS19;CK2α'^(+/−)^ p=0.0212, PS19 vs. CK2α'^(+/−)^ p<0.0001, I: WT vs. PS19 p=0.0017, PS19 vs. CK2α'^(+/−)^ p=0.0004) **(J)** Experimental set-up: R, recording electrode; S stimulating electrode. **(K)** Representative traces before (1) and after (2) Theta-burst stimulation (TBS). **(L)** Course temporal of TBS application inducing LTP in pyramidal neurons of CA1 (n=6-7 mice/genotype). **(M)** Short-term potentiation (STP) results, significance reported relative to WT (WT vs. PS19 p=0.014, WT vs. PS19;CK2α’^(+/−)^ p=0.007). **(N)** Input-output curves. Averaged fEPSP amplitude, (n=3mice/genotype). **(O)** Long-term potentiation (LTP) results, significance reported relative to WT (WT vs. PS19 p=0.007). All data are shown as mean ± SEM. Statistics: one way-ANOVA with Tukey’s (**B**), repeated measures ANOVA with Geisser-greenhouse correction and Tukey’s (**D-I**), or one-way ANOVA with Holm-Sidak (**M-O**). Significance displayed as compact letter display or *p<0.05, **p<0.01, ***p<0.001, ****p<0.0001.

We conducted NeuN immunostaining analyses in the hippocampus of PS19 and PS19;CK2α’^(+/−)^ mice to determine if depletion of CK2α’ could impact the overall neuronal loss characteristic of PS19 mice [40, 74-76]. Significant depletion of NeuN + cells and hippocampal atrophy was only seen in PS19 groups at a symptomatic age (Fig. 5A, B, **Supp. Figure 4A-C**). An ~ 30–50% reduction in the number of NeuN counts is often reported at late symptomatic ages in PS19 mice [40, 74-76]. However, we noticed a great variability in the number of NeuN + cells and hippocampal area among PS19 littermates at this disease stage that was not related to sex (**Supp. Figure 4D-F**). Wide variability in tau pathology and neuronal degeneration within this mouse model has been previously reported and associated to nuances in the genetics of this model [77]. Therefore, we categorized PS19 and PS19;CK2α’^(+/−)^ animals presenting NeuN counts within 10% of the WT as “abnormally low pathology” and as a result 3 PS19 animals and 1 PS19;CK2α’^(+/−)^ were excluded from subsequent analyses. Analyses in curated groups of PS19 and PS19;CK2α’^(+/−)^ mice revealed a significant increase in the number of NeuN + cells in PS19;CK2α’^(+/−)^ mice compared to PS19 in the CA1 (Fig. 5A, B, **Supp. Figure 4A-C**).

We then evaluated if total synapse density was also positively altered in the CA1. We conducted immunostaining and colocalization analyses for the vesicular glutamate transporter 1 (VGlut1) (pre-synaptic marker of excitatory synapses), and the postsynaptic density protein 95 (PSD95) post-synaptic marker) (Fig. 5C-I). As expected, PS19 mice showed a significant decrease in the number of synapses (colocalized puncta VGlut1-PSD95) compared to WT mice (Fig. 5F, I). The reduction in colocalized puncta in PS19 mice was due to a concomitant reduction in both PSD95 and VGlut1 markers (Fig. 5D, E, G, H). In PS19;CK2α’^(+/−)^ mice we found that although there is not a statistical significance compared to PS19 mice, CK2α’ haploinsufficiency decreased the % loss of both PSD95 (Fig. 5D, G) and VGlut1 (Fig. 5E, H). This resulted in the amelioration of total synapse loss in PS19;CK2α’^(+/−)^ mice and obtained values for colocalization were no longer significant respect to WT (Fig. 5F, I). These results demonstrate a partial rescue in the density of excitatory synapses in the CA1 of PS19;CK2α’^(+/−)^ mice aligning with the amelioration of hippocampal atrophy observed in this region (Fig. 5B).

To investigate functional implications of the rescue seen in CA1 hippocampal atrophy and synaptic density we assessed hippocampal synaptic plasticity by performing extracellular field excitatory postsynaptic potential (fEPSP) recordings in the CA1 region of acute hippocampal slices (Fig. 5J, K). Theta-burst stimulation (TBS) at the Schaffer collaterals induced short-term potentiation (STP), which resulted in a significant increase in synaptic efficacy at CA1 synapses in both WT and CK2α’^(+/−)^ mice, as evidenced by the enhanced fEPSP amplitude (170 ± 13%, n = 6; 170 ± 12%, n = 7) (Fig. 5L, M). In contrast, the increase in fEPSP amplitude was significantly reduced in PS19 and PS19;CK2α’^(+/−)^ mice relative to WT controls post TBS (123 ± 4%, n = 7; 128 ± 7%,n = 6; Fig. 5L, M), indicating impaired STP in these models. Furthermore, we examined long-term potentiation (LTP) at CA3-CA1 synapses, a form of synaptic plasticity linked to learning and memory. It has been previously shown that PS19 mice present impaired LTP in the CA1-CA3 pathway [40, 78, 79]. First, we observed significant differences in input-output curves at CA3-CA1 synapses between PS19 and PS19;CK2α’^(+/−)^ mice when compared to WT, which suggests that excitatory synaptic input properties were altered in these models (Fig. 5N). TBS at the Schaffer collaterals induced robust LTP in both WT (173 ± 12%, n = 6) and CK2α’^(+/−)^ (164 ± 9%, n = 7; Fig. 5L, O). However, in the PS19 mouse model, the magnitude of LTP was significantly diminished compared to WT (128 ± 9%, n = 7; vs. WT p = 0.007) (Fig. 5L, O), suggesting a disruption in the molecular mechanisms underlying LTP. Interestingly, in the PS19;CK2α’^(+/−)^ mice, LTP was partially restored (150 ± 6%, n = 6; vs. WT p = 0.199) (Fig. 5L, O), pointing to a potential compensatory mechanism that partially mitigates the LTP deficits observed in the PS19 mice. Importantly, paired-pulse facilitation (PPF) was unaltered across all experimental groups, suggesting that the observed changes in synaptic plasticity were most likely due to postsynaptic mechanisms dependent on the hippocampus (**Supp**. Figure 5).

### CK2α’ haploinsufficiency improved PS19 behavior on Barnes Maze

**Figure 6:**
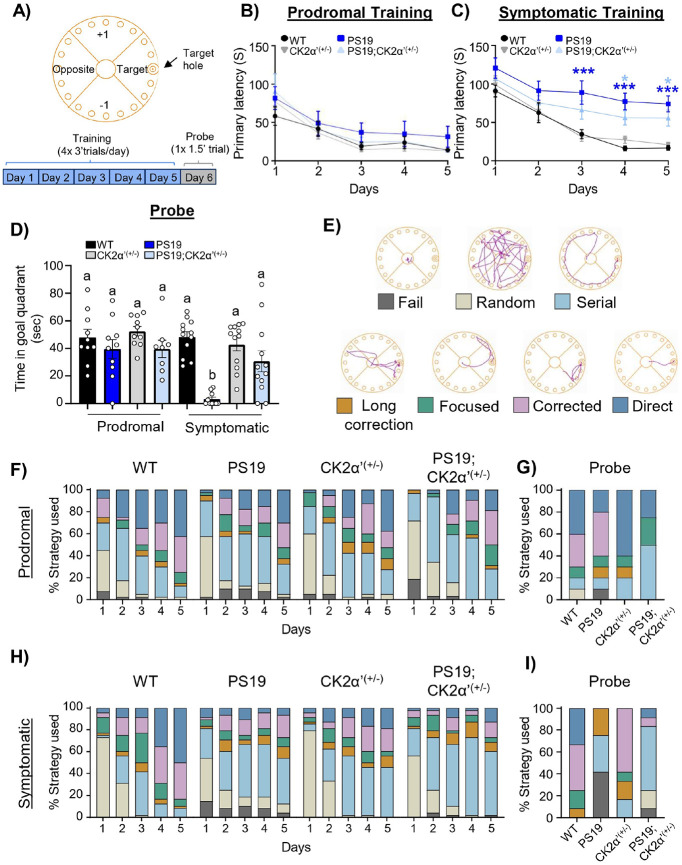
Depletion of CK2α’ improved spatial memory of PS19 mice on the Barnes maze. **(A)**Schematic of the Barnes Maze divided into 4 quadrants (Target, Opposite, +1 and −1) and experimental set up. **(B-C)** Primary latency (s), or time to first explore escape hole at prodromal time point. Statistical analyses were conducted using two-way ANOVA with Bonferroni’s multiple comparison’s. Prodromal; genotype p=0.0573, training day p<0.0001 and interaction p=0.9690. Symptomatic: genotype p<0.0001, training day p<0.0001 and interaction p=0.7018. Differences for each training day were conducted using Bonferroni’s multiple comparisons and * show significance relative to WT. **(D)** Time spent in goal quadrant of Barnes maze during probe trial at prodromal and symptomatic time points. n=10-12 mice/genotype, 2 outliers were removed from PS19 group using ROUT (Q=1%). **(E)** Representative search strategy traces based on their navigation patterns on the maze. **(F,H)** Spatial strategies used to locate the escape hole across training days for each genotype at prodromal and symptomatic stages, respectively. **G,I)** Spatial strategies used to locate the previously learned escape target hole during probe trial. Data are shown as mean ± SEM. Statistical analyses were conducted using Two-way ANOVA with Bonferroni’s multiple comparisons (B-C) or Two-Way ANOVA with Tukey’s post-hoc analysis (D). Significance displayed with either *p<0.05, **p<0.01, ***p<0.001, ****p<0.0001 or compact letter display.

We then tested whether the partial rescue observed in PS19;CK2α’^(+/−)^ mice associated to synaptic density and functional LTP along with the decrease in Tau pathology had any impact in cognitive behaviors. Mice were trained on the Barnes maze task for 5 consecutive days on the location of a target hole (training) and on the 6th day a probe trial was conducted with the target hole removed (Fig. 6A). During the training trials the primary latency or the time to first locate the escape hole was recorded (Fig. 6B-C). All mice in the prodromal group demonstrated an improvement on time needed to locate the escape hole from day 1 vs day 5 (WT p = 0.0272; PS19 p = 0.0.0067; CK2α’^(+/−)^ p = 0.0002; PS19;CK2α’^(+/−)^ p < 0.0001) with no significant difference between the genotypes (Fig. 6B). All mice in the symptomatic group also showed a capability of acquiring the task demonstrating a significant improvement from day 1 vs day 5 (WT p < 0.0001; PS19 p = 0.0109; CK2α’^(+/−)^ p < 0.0001; PS19;CK2α’^(+/−)^ p = 0.0037). However, PS19 and PS19;CK2α’^(+/−)^ showed a significant impairment compared to WT, starting on day 3 for PS19 (p = 0.0009) and day 4 for PS19;CK2α’^(+/−)^ (p = 0.0306) (Fig. 6C). The training data suggested that both symptomatic PS19 and PS19;CK2α’^(+/−)^ mice showed similar impaired learning on the Barnes Maze compared to WT mice, although deficits in PS19;CK2α’^(+/−)^ were attenuated.

Following the training phase of the Barnes maze we conducted a probe phase in which the escape hole was covered and the time spent exploring the escape hole quadrant was recorded. At the prodromal age there was no significant difference in time spent in the escape hole quadrant for any genotype (Fig. 6D). At the symptomatic age, the PS19 mice showed a significant impairment in the time spent in the goal quadrant compared to WT (p < 0.0001) (Fig. 6D). Importantly, the PS19;CK2α’^(+/−)^ mice spent significantly more time than the PS19 mice in the goal quadrant with no significant difference from WT (vs. PS19 p = 0.0113 vs. WT p = 0.2335) demonstrating a rescue in spatial memory for the PS19;CK2α’^(+/−)^ on the probe phase of the Barnes maze (Fig. 6D).

The PS19 mouse model is known to present motor deficits due to hindlimb paralysis and weakness[40], which could confound Barnes Maze results if PS19 mice perform more poorly due to less distance traveled. Prodromal mice displayed no difference in distance traveled on Barnes Maze (**Supp. Figure 6A**). While the symptomatic PS19 mice traveled less distance in the Barnes Maze compared to WT mice, they did not show a significant difference in distance traveled compared to PS19;CK2α’^(+/−)^ mice (**Supp. Figure 6B**). In addition, in open-field testing we found no differences between groups at the prodromal time point (**Supp. Figure 6C**). On the contrary, symptomatic mice showed significant hyperactivity in the open field task, demonstrating the ability of PS19 mice to move (**Supp. Figure 6D**). Indeed, no significant differences were observed in the open field between PS19 and PS19;CK2α’^(+/−)^ mice (**Supp. Figure 6D**). This data supports the improvement in cognitive abilities seen in the PS19;CK2α’^(+/−)^ mice are not due to changes in motor function but rather are related to an improvement in memory.

Mice can use different cognitive strategies to navigate the Barnes Maze and locate the escape hole [57]. As the mouse learns the task they show improvement in the strategy used to locate the escape hole [57] (Fig. 6E). Assessing these cognitive strategies is important to provide a deeper insight into how memory and spatial learning are being utilized. All animals in the prodromal group demonstrated a similar pattern in spatial strategies over the training phase (Fig. 6F) and showed a predominance in the corrected and direct strategies in the probe phase (Fig. 6G). Symptomatic PS19 and PS19;CK2α’^(+/−)^ mice both showed impaired spatial strategies over the training phase, by day 5 both genotypes largely displayed either failing, random or serial strategies (Fig. 6H). However, during the shortened probe phase, PS19 displayed a worsened overall strategy, with predominant failing, serial, and long correction strategies compared to PS19;CK2α’^(+/−)^ mice that displayed more serial, corrected and direct strategies (Fig. 6I). Overall, the PS19;CK2α’^(+/−)^ mice displayed improvement in the probe phase of the Barnes Maze in both the time spent in the escape quadrant and strategy used to locate the target, indicating improvements in spatial memory.

### RNA-Seq analyses revealed CK2α’ alters the expression of genes related to immune response and synaptic function

To start interrogating the mechanisms by which CK2α’ influences tau pathology and cognitive decline in PS19 mice we conducted RNA-Seq analysis in the hippocampus of animals in the prodromal phase. The reason we focused on this particular age group is because the prodromal phase represents the initial stage of disease development that precedes overt symptoms. During this time, sometimes subtle but significant molecular changes begin to occur, including alterations in gene expression that precede the onset of full-blown pathology. By focusing on this phase, we aimed to identify pathways involved in disease onset that may be missed if only later stages of the disease are studied where significant neuronal loss is present. Our RNA-seq analyses revealed a large variability in the expression pattern of PS19 mice that associated to the variability observed in NeuN counts (**Supp. Figure 4D-F**). By comparing the expression pattern of the different animals along with the tau pathology we found two distinct expression profiles that allowed the mice to segregate into low (Low-PS19) and high (High-PS19) pathology groups (**Supp. Figure 7A**). These two different pathology groups showed differential gene expression compared with WT from one another (**Supp. Figure 7B-C**). With the Low-PS19 group primarily differentially dysregulated genes (DGEs) related to developmental and organizational pathways (**Supp. Figure 7B**) and the High-PS19 showing DGEs related to immune processes (**Supp. Figure 7C**). Interestingly, the High-PS19 group was more similar in the gene expression pattern to the PS19;CK2α’^(+/−)^ group than the Low-PS19 group (**Supp. Figure 7D-E**). Moreover, the PS19;CK2α’^(+/−)^ mice showed a more similar expression pattern among animals of the same group. Moving forward we focused our comparisons on the High-PS19 group with PS19;CK2α’^(+/−)^.

**Figure 7:**
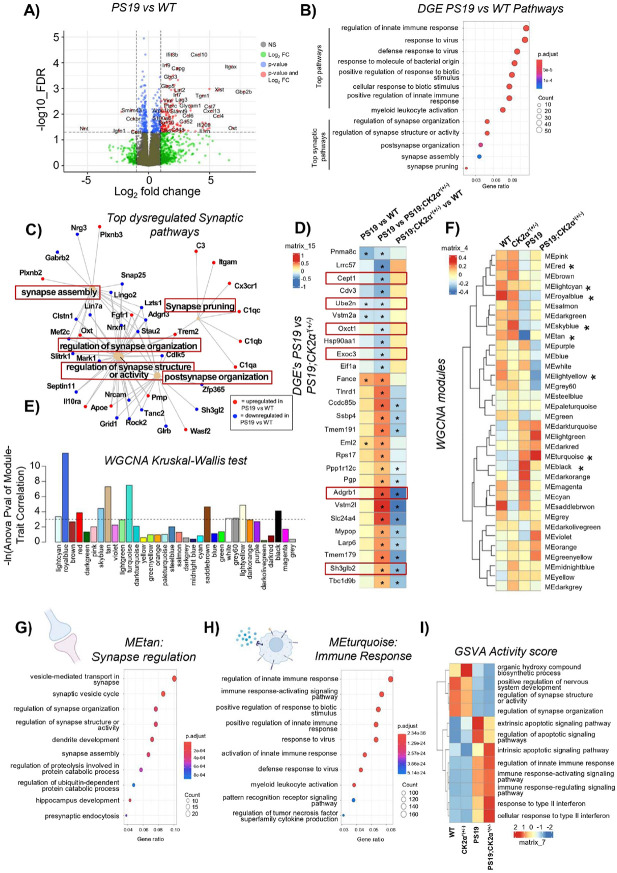
CK2α’ haploinsufficiency has transcriptional impacts on synapses and immune related gene pathways in PS19 mice. **(A)** Volcano plot of DGEs in PS19 vs WT comparison (n=3-4 mice/genotype). **(B)** Geneontology top biological pathways for DGEs in PS19 vs WT. **(C)** Gene network for top 5 synaptic pathways impacted in PS19 vs WT. Red dots indicate genes upregulated in PS19 mice. Blue dots indicate genes down-regulated in PS19 mice. **(D)** Heat map displaying average FC across three comparisons PS19 vs WT, PS19 vs PS19; CK2α’^(+/−)^ and PS19; CK2α’^(+/−)^ vs WT for all significant DGEs identified via PS19 vs PS19; CK2α’^(+/−)^ comparison. Red boxes indicate genes with apoptotic/phagocytotic and or immune-related functions. *q>0.05. **(E)** WGCNA identified modules, dotted line indicates significance as determined by the Kruskal wallis test between WT, CK2α’^(+/−)^, PS19, and PS19;CK2α’^(+/−)^ mice (n=3-5 mice/genotype). **(F)** Heatmap of modules detected from WGCNA indicating differences across genotypes. * indicates p<0.05 determined by Kruskal-wallis test. **(G, H)** Gene ontology for top biological pathways for MEtan module (**G**) and MEturquoise module (**H**). **(I)** Average representation of pathway activity scores determined via GSVA analysis. Panels **G** and **H** were created in BioRender (White, A. (2025) https://BioRender.com/eqznm6m).

Gene expression analyses in the High-PS19 cohort (referred hereafter as PS19) vs WT mice identified 477 significant DGEs in the PS19 vs WT (Fig. 7A). Gene ontology pathway analysis revealed that many of the DGEs belonging to the top dysregulated pathways corresponded to immune and inflammatory functions (Fig. 7B), as previously reported [80, 81]. Other important GO pathways were associated with synaptic dysfunction where the top 5 impacted synaptic pathways in PS19 mice corresponded to dysregulation in synapse assembly, regulation of synapse organization, regulation of synapse structure or activity, post synapse organization and synaptic pruning (Fig. 7B-C). Synaptic pruning was upregulated in PS19 vs WT, whereas the other synaptic pathways related to organization and regulation of activity were largely downregulated (Fig. 7C). These results aligned with the decreased synapse density and impaired synaptic function seen in PS19 mice and indicated a potential early prodromal transcriptional signature for these deficits.

**Figure 8:**
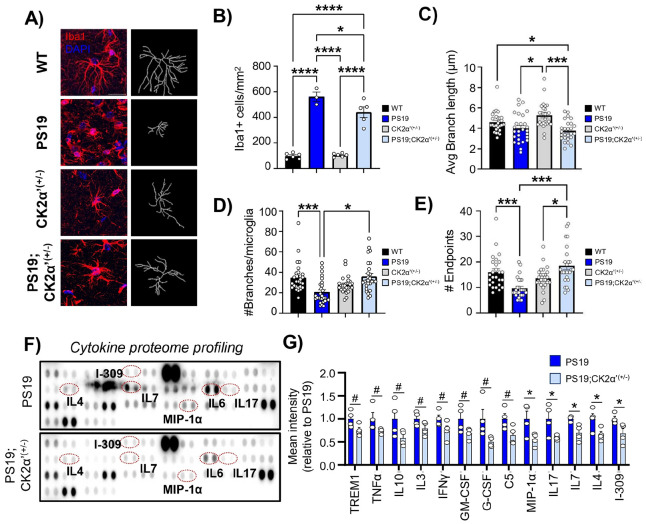
CK2α’ haploinsufficiency impacts Iba1+ microglia morphology and cytokine production in PS19 mice. **(A)** Iba1+ microglia immunostaining and microglia skeleton representation from the CA1 of symptomatic animals used for microglia morphology analysis. **(B)** Quantification of the number of Iba1+ positive cells in the CA1 (n=3-6 mice/genotype; WT vs. PS19 p<0.0001, WT vs. CK2α'^(+/−)^ p=0.9963, WT vs. PS19; CK2α'^(+/−)^ p<0.0001, PS19 vs. CK2α'^(+/−)^ p<0.0001, PS19 vs. PS19;CK2α'^(+/−)^ p=0.0313, CK2α'^(+/−)^ vs. PS19;CK2α'^(+/−)^ p<0.0001). **(C)** Quantification of microglia branch length (WT vs. PS19 p=0.4402, WT vs. CK2α'^(+/−)^ p=0.1371, WT vs. PS19; CK2α'^(+/−)^ p=0.041, PS19 vs. CK2α'^(+/−)^ p=0.0184, PS19 vs. PS19;CK2α'^(+/−)^ p=0.927, CK2α'^(+/−)^ vs. PS19;CK2α'^(+/−)^ p=0.0004), **(D)** the number of branches per microglia (WT vs. PS19 p=0.0008, WT vs. CK2α'^(+/−)^ p=0.2116, WT vs. PS19;CK2α'^(+/−)^ p=0.9908, PS19 vs. CK2α'^(+/−)^ p=0.3063, PS19 vs. PS19;CK2α'^(+/−)^ p=0.0114, CK2α'^(+/−)^ vs. PS19; CK2α'^(+/−)^ p=0.0757), **(E)** and number of endpoints per microglia (WT vs. PS19 p=0.0003, WT vs. CK2α'^(+/−)^ p=0.3541, WT vs. PS19;CK2α'^(+/−)^ p=0.5111, PS19 vs. CK2α'^(+/−)^ p=0.0556, PS19 vs. PS19;CK2α'^(+/−)^ p=0.0004, CK2α'^(+/−)^ vs. PS19;CK2α'^(+/−)^ p=0.0179) (n=27 cells (3 mice/genotype, 3 slices/mouse, 3 cells/slice). (F) Representative cytokine arrays from PS19 and PS19;CK2α’^(+/−)^ at 9 months from hippocampal protein extracts. Cytokines that showed significant differences are circled in red. **(G)** Selected quantifications of cytokines showing significance or trending significance. Quantifications are represented relative to WT (n=4 mice/genotype;Trem1 p=0.0624, Tnfa p=0.1009, IL10 p=0.0948, IL3 p=0.0952, IFNy p=0.0972, GM-CSF p=0.0816, G-CSF p=0.0562, C5 p=0.0522, Mip 1a p=0.0482, IL17 p=0.0386, IL7 p=0.0481, IL4 p=0.046, I309 p=0.039). Data are shown as mean ± SEM. Statistical analyses were conducted using ANOVA with Geisser-greenhouse correction and Tukey's post-hoc analysis in **B-E,** and unpaired t-test in **G.** #p≤0.1, *p<0.05, **p<0.01, ***p<0.001, ****p<0.0001.

Direct comparison of PS19 vs PS19;CK2α’^(+/−)^ mice yield a small set of 26 significant DGEs (Fig. 7D). This small gene set returned no biologically connected pathways via gene ontology analysis, but several of the DGEs were related to Apoptotic/phagocytic and Immune/inflammatory functions (*Cept1, Ube2n, Oxct1, Exoc3, Adgrb1, Sh3glb2*, *Adgrdb1*). We then assessed whether the manipulation of CK2α’ had any overall effect on modules of genes with similar biological functions despite not reaching the > Log2 criteria. Weighted Gene Co-Expression Network Analysis (WGNCA) revealed 34 gene modules with 8 modules (red, lightcyan, royalblue, skyblue, tan, lightyellow, turquoise, and black) showing statistical significance detected via a Kruskal-Wallis test among our groups (p < 0.05) (Fig. 7E, F, **Supp. File 3**). Interestingly several of these modules (red, tan, and black) showed a trend where the PS19;CK2α’^(+/−)^ mice more closely resembled the expression pattern of WT mice. On the contrary, other modules showed a larger dysregulation in the PS19;CK2α’^(+/−)^ compared to WT and PS19, such as the lightcyan, lightyellow, and turquoise. Among the 8 significant gene modules, we focused our analyses on the tan and turquoise modules for showing a different expression pattern between PS19 and PS19;CK2α’^(+/−)^ mice. The tan module composed on 215 genes (Kruskal-wallis p = 0.016) (Fig. 7F-G, **Supp. Figure 8A, Supp. File 3**) was represented by pathways associated with synaptic function and assembly, with top pathways being vesicle-mediated transport in synapse, synaptic vesicle cycle, and regulation of synapse organization (Fig. 8G). The overall expression of genes associated with these pathways presented a depletion in the PS19 mice that was ameliorated in the PS19;CK2α’^(+/−)^ group (Fig. 7F). Changes in these pathways aligned with the improvements observed in NeuN abundance, synapse density and function in PS19;CK2α’^(+/−)^ mice (Fig. 5).

On the other hand, the turquoise module composed of 2765 genes (Kruskal-wallis p = 0.0041) displayed a dysregulation in the same direction in both PS19 and PS19;CK2α’^(+/−)^ mice, with a greater dysregulation in PS19;CK2α’^(+/−)^ (Fig. 7F, H, **Supp. Figure 8B, Supp. File 3**). This module was a large collection of genes including top significant pathways related to innate immune response and immune response-activating signaling pathways (Fig. 7H). PS19;CK2α’^(+/−)^ mice showed a higher expression than PS19 of a large set genes associated with immune response perhaps indicating an improved activation of immune systems.

To further explore DGEs specific to the PS19 and PS19;CK2α’^(+/−)^ mice we focused specifically on their comparisons to WT. We set criteria to yield two comparisons (*comparison 1*) significant DGEs in PS19 vs WT but not PS19;CK2α’^(+/−)^ vs WT and (*comparison 2*) significant DGEs in PS19;CK2α’^(+/−)^ vs WT but not PS19 vs WT. This more restrictive criteria then yielded two unique set of genes (**Supp. File 4**). Biological pathways associated with *comparison 1* included regulation of neurogenesis, positive regulation of nervous system development and regulation of synapse organization (**Supp. Figure 8C**), similar to those pathways identified in the tan module. Biological pathways associated with *comparison 2* included those associated with inflammatory or immune responses (**Supp.** Figure 8D). The majority of genes included in the top two biological pathways were upregulated in PS19;CK2α’^(+/−)^ mice and associated with immune response (**Supp. Figure 8D, E**). These results indicate there are more immune-related genes significantly differentially expressed in the PS19;CK2α’^(+/−)^ compared to WT than PS19 compared to WT similar to what was revealed in the turquoise module (Fig. 7H). This was also confirmed by gene set variation analysis (GSVA) activity scores of the dysregulated pathways within *comparison 2* showing increased activity of pathways associated with immune response relative to WT (Fig. 7I). This analysis also revealed interesting alterations in apoptotic pathways. We observed a decrease in activity in extrinsic apoptotic pathways and an increase in intrinsic apoptotic pathways in the PS19;CK2α’^(+/−)^ when comparing to PS19 mice (Fig. 7I). This suggests a shift in the molecular mechanisms governing the apoptotic pathway towards a more beneficial and selective apoptotic environment perhaps contributing to the beneficial outcomes observed in PS19;CK2α’^(+/−)^ mice. Overall, the RNA-Seq analyses revealed that CK2α’ haploinsufficiency caused alterations in networks of genes associated with important and relevant biological pathways demonstrating the amelioration of synaptic dysfunction, the enhanced activation of immune related pathways, and impacts on apoptotic signaling.

### PS19 mice lacking CK2α’ ameliorated microglia reactivity, neuroinflammation and phagocytic activity

Considering the enhanced levels of CK2α’ in microglia, the activation of pathways involved in immune response upon deletion of CK2α’, and the key role of microglia as CNS immune cells in tauopathies and dementia [12, 40, 82-85], we examined the state of glial cells (astrocytes and microglia) in the hippocampus in both the prodromal and symptomatic mice groups. First, we observed a significant increase in the number of GFAP + cells in the hippocampus of PS19 mice in both age groups (**Supp. Figure 9A-E**), as previously reported [40], but we did not find any differences compared to PS19;CK2α’^(+/−)^ mice (**Supp. Figure 9C-E**). However, GFAP + cells significantly decreased in the PS19;CK2α’^(+/−)^ compared to the PS19 mice in the overlaying cortex of symptomatic mice (**Supp. Figure 9F-H**). When looking at Iba1 + cells we observed a modest but significant increase in Iba1 + positive cells throughout the hippocampus in the prodromal PS19 mice compared to WT (**Supp. Figure 10A, C-E**). In the symptomatic group, both the PS19 and PS19;CK2α’^(+/−)^ demonstrated a much greater increase in the number of Iba1 + cells compared to WT, but PS19;CK2α’^(+/−)^ mice presented significantly less Iba1 + cells than PS19 in both the CA1 (Fig. 8A, B, **Supp. Figure 10B, C**) and CA3 (**Supp. Figure 10B, E**). No significant differences were observed among the PS19 in the DG (**Supp. Figure 10B, E**). We also observed significantly less Iba1 + cells in the PS19;CK2α’^(+/−)^ compared to PS19 mice in the overlaying cortex of symptomatic mice (**Supp. Figure 10F-H**).

We also examined microglia morphology using measurements corresponding to ramification and correlating with microglia state (Fig. 8A, C-E). It has been previously reported that microglia adopt an amoeboid morphology in PS19 mice, characterized by the loss of microglia processes, and that is associated with a more reactive and phagocytic state [12, 86]. We examined microglia morphology in the CA1 in symptomatic mice as we observed the largest decrease in Iba1 + cell counts in this hippocampal region (Fig. 8B, **Supp. 10C-E**). Both the PS19 and PS19;CK2α’^(+/−)^ mice showed a decrease in the average branch length (Fig. 8C). However, PS19;CK2α’^(+/−)^ mice demonstrated a significant increase in the number of branches and number of endpoints compared to the PS19 mice (Fig. 8D, E) indicating an improvement in the morphological state of microglia.

**Figure 9:**
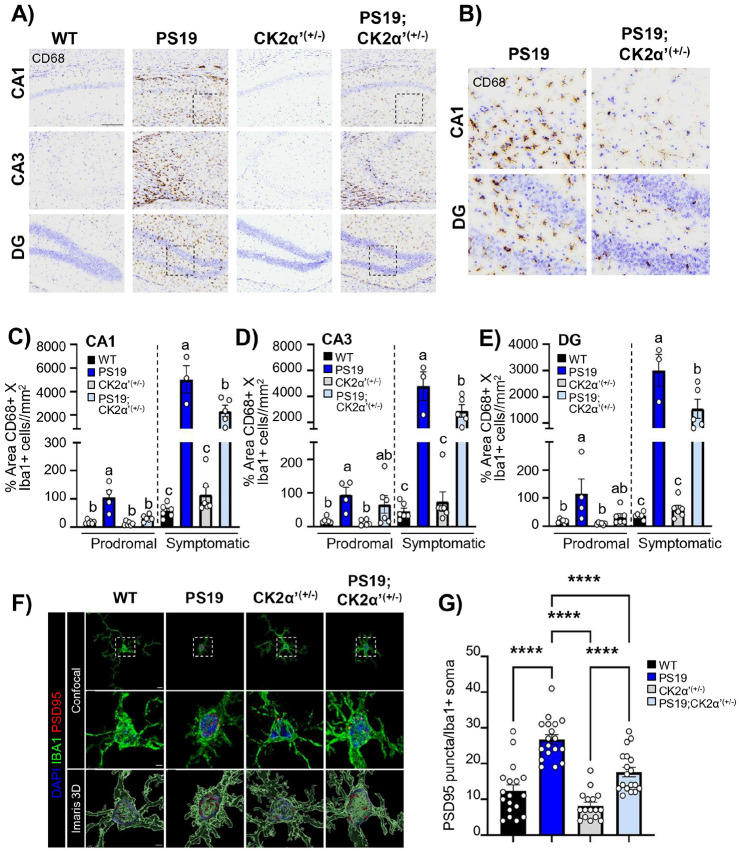
CK2α’ haploinsufficiency decreases microglia reactivity and synaptic engulfment. **(A)** CD68 immunostaining with cresyl violet counterstain in the hippocampus of symptomatic mice. Scale bar=150 μm. (**B**) zoomed images from A in the CA1 and DG. **(C-E)** Quantifications of % area CD68+ normalized by Iba1+ cell count in prodromal and symptomatic cohorts in CA1, CA3 and DG (n=3-6 mice/genotype). Comparisons shown relative to cohort age group (**CA1:** *Prodromal:* WT vs. PS19 p<0.0001, PS19 vs. CK2α'^(+/−)^ p<0.0001, PS19 vs. PS19;CK2α'^(+/−)^ p=0.0003, *Symptomatic:* WT vs. PS19 p<0.0001, WT vs. PS19;CK2α'^(+/−)^ p=0.0036, PS19 vs. CK2α'^(+/−)^ p<0.0001, PS19 vs. PS19;CK2α'^(+/−)^ p=0.0042, CK2a'(+/−) vs. PS19;CK2α'^(+/−)^ p=0.0045 **CA3:** *Prodromal:* WT vs. PS19 p=0.0328, WT vs. PS19;CK2α'^(+/−)^ p=0.1739, PS19 vs. CK2α'^(+/−)^ p=0.0193, PS19 vs. PS19; CK2α'^(+/−)^ p=0.6895, CK2α'(+/−) vs. PS19;CK2α'^(+/−)^ p=0.1045 *Symptomatic:* WT vs. PS19 p<0.0001, WT vs. PS19;CK2α'^(+/−)^ p=0.0003, PS19 vs. CK2α'^(+/−)^ p<0.0001, PS19 vs. PS19;CK2α'^(+/−)^ p=0.0404, CK2α'^(+/−)^ vs. PS19; CK2α'^(+/−)^ p=0.0003 **DG:** *Prodromal:* WT vs. PS19 p=0.0176, WT vs. PS19; CK2α'^(+/−)^ p=0.9281, PS19 vs. CK2α'^(+/−)^ p=0.0091, PS19 vs. PS19;CK2α'^(+/−)^ p=0.0536, CK2α'^(+/−)^ vs. PS19;CK2α'^(+/−)^ p=0.7762 *Symptomatic:* WT vs. PS19 p<0.0001, WT vs. PS19;CK2α'^(+/−)^ p=0.0018, PS19 vs. CK2α'^(+/−)^ p<0.0001, PS19 vs. PS19;CK2α'^(+/−)^ p=0.011, CK2α'^(+/−)^ vs. PS19; CK2α'^(+/−)^ p=0.002. **(F)** Iba1 and PSD95 immunostaining from the CA1 of symptomatic animals. Top row: whole microglia including processes, scale bar= 5μm. Middle row: higher magnification view of the soma (white dotted box in top row), scale bar= 2μm. Bottom row: 3D reconstruction of Iba1 and PSD95 signal generated from Imaris Software, scale bar= 2μm. **(G)** Quantification of the number of PSD95 puncta/Iba1+ soma (n= 16-18 cell, 3 mice/genotype, 2-3 slices/mouse, 22 cells/slice; WT vs. PS19 p<0.0001, WT vs. PS19; CK2α'^(+/−)^ p=0.0854, PS19 vs. CK2α'^(+/−)^ p<0.0001, PS19 vs. PS19; CK2α'^(+/−)^ p=0.0025, CK2α'^(+/−)^ vs. PS19;CK2α'^(+/−)^ p=0.0208). Data are show as mean ± SEM. Statistical analyses were conducted using one-way ANOVA with Tukey’s post-hoc *p<0.05, **p<0.01, ***p<0.001, ****p<0.0001.

Since the morphological state of microglia is strongly related to its reactive state and neuroinflammation we first assessed the expression of a variety of cytokines and other inflammatory molecules associated with microglia reactivity by using a cytokine array panel with hippocampal extracts from symptomatic PS19 and PS19;CK2α’^(+/−)^ (Fig. 8F, G). Importantly, we confirmed that several cytokines were significantly decreased (p < 0.05) in PS19;CK2α’^(+/−)^ mice relative to PS19 including, MIP-1α, IL17, IL7, IL4, and I309 with several others showing trending decreases (0.05 < p < 0.1) including TREM1 and TNFα (Fig. 8F, G). We also investigated whether changes in overall cytokine levels and microglia morphology upon CK2α’ depletion related to an altered microglia reactive state. For this we assessed the levels of CD68 in the hippocampus of mice and normalized to the number of Iba1 + cells observed in each animal and time point (Fig. 9A-E, **Supp. Figure 10I**). We observed increased levels of CD68 in all regions of the hippocampus in the PS19 mice at both prodromal and symptomatic stages (Fig. 9C-E). PS19;CK2α’^(+/−)^ mice also presented increased CD68 levels compared to WT but displayed significantly less CD68 in the CA1 and a trending decrease in the CA3 and DG at the prodromal stage compared to PS19 mice (Fig. 9C-E). Importantly, symptomatic PS19;CK2α’^(+/−)^ mice displayed significantly less CD68 in all hippocampal regions compared to PS19 mice (Fig. 9C-E).

Reactive microglia have also been implicated in synaptic and neuronal loss in tauopathy [80, 87-91]. Prior studies have demonstrated that reactivity state of microglia is associated with microglial engulfment of synapses which can be assessed through colocalization of synaptic markers such as PSD95 within microglia [92, 93]. To quantify synapse phagocytosis/engulfment we co-stained for PSD95 and Iba1 and examined the CA1 stratum radiatum of symptomatic mice (Fig. 9F). PSD95 + puncta were quantified within the Iba1 + cell soma, which is considered to be lysosomes containing degraded material are present [94, 95]. PS19 mice exhibited a significant increase in PSD95 + puncta within Iba1 + relative to control mice (Fig. 9F, G), demonstrating increased phagocytosis in PS19 microglia. Importantly, PS19;CK2α’^(+/−)^ showed a significant reduction in PSD95 + engulfment (Fig. 9F, G) indicating an amelioration in the phagocytic activity of microglia upon deletion of CK2α’.

## Discussion

Tauopathies are a heterogeneous group of neurodegenerative disorders for which effective treatments are still not available, in part due to an incomplete understanding of the underlying mechanisms. In this study, we present a novel target, CK2α’, a catalytic subunit of CK2, as a potential upstream regulator of tau mediated neurodegeneration, acting at the intersection of immune signaling and synaptic dysfunction.

Here, we report that CK2α’ expression is abnormally elevated in the brains of dementia patients and in the hippocampus of PS19 mice, with preferential upregulation observed in hippocampal neurons and microglia. These findings are supported by multiple single-cell RNA-sequencing (snRNA-seq) studies in human AD, which consistently show increased CK2α’ expression in excitatory neurons [69-71]. Additionally, the study by Gerrits et al. [68] in the occipital lobe (OL) has reported preferential CK2α’ expression in AD-associated microglia. Other scRNA-seq studies have detected elevated CK2α’ expression in various brain cell types, with patterns varying by dataset and brain region [69-71], while a recently published study has shown that CK2α is increased in cortical astrocytes of the 5xFAD and PS19 models [96]. Although these datasets lack specific information on the hippocampus, the collective evidence suggests that CK2α’ dysregulation in AD/ADRD may be both cell-type- and region-specific.

Increased CK2 levels and/or activity have been previously reported in AD and AD models [19, 37, 72], and CK2 has been associated with tau pathology [19, 20, 72, 97], microglia state [98, 99] and general inflammation [100-106]. However, many of these studies were conducted in cell models [19, 20, 37, 72] and genetic evidence for the specific contributions of the individual CK2 subunits to these processes remained largely unexplored. Our studies in vitro using N2A cells transfected with Tau-P301L showed that silencing CK2α’, but not CK2α, decreased pTau accumulation. Furthermore, our studies in vivo reducing the levels of CK2α’ in the PS19 model also influenced tau pathology by decreasing the accumulation of pTau and tau burden in the hippocampus and cortex of PS19;CK2α’^(+/−)^ mice. In support to our data, previous studies *in vitro* by Perez et al. showed that overexpression of CK2α’ subunit decreased the activity of I2PP2A/SET, a cellular protein that functions as an endogenous inhibitor of protein phosphatase 2A (PP2A), increasing pTau. In contrast overexpression of CK2α increased the activity of I2PPA2a/SET [97]. Although early studies demonstrated that Tau is a physiological substrate of CK2, at least in specific contexts such as neurogenesis [107], the findings by Perez et al. [97] suggest that the effects we observed after manipulating CK2α’ on pTau accumulation could be mediated through intermediary molecules that modulate Tau phosphorylation and not directly.

Haploinsufficiency of CK2α’ also increased the expression of synaptic genes, synaptic density, and synaptic function in the PS19 mice. Along these lines, haploinsufficiency of CK2α’ in a mouse model of HD also showed improved synaptic density and function [18, 54], although how exactly CK2α’ influences these processes is still unknown. A previous study showed that CK2α’ can influence the expression of synaptic genes, especially those related to glutamatergic excitatory signaling [18]. A more recent study showed that pharmacological inhibition of CK2 mitigated AD tau pathology by preventing the NMDA receptor subunit NR2B synaptic mislocalization [72]. NR2B mediates long-term depression (LTD), and LTD specifically induces the phosphorylation of tau. These studies support our findings and strengthen the relationship between CK2α’, tau phosphorylation, and the regulation of neuronal function in both AD and other neurodegenerative diseases. However, since CK2α’ is found elevated in both neurons and microglia, it is still unknown whether the effects mediated by CK2α’ in pTau accumulation and synaptic function are cell or non-cell autonomous, warranting further investigation.

CK2 has been identified as a mediator of microglia reactivity and inflammation in different models [98, 99] and it has been well-established as a mediator of inflammation in a variety of contexts including SARS-CoV infection, cancers, bacterial infections, intestinal inflammation and renal failure [100-105]. This is largely mediated via CK2’s involvement in regulating several large inflammatory factors such as NF-κB, STAT1, and EGR-1 [106]. It is known that chronic activation of NF-kB increases the accumulation of pTau [12]. On the other hand, pathological tau has also been shown to induce inflammation and NF-kB activation by interacting directly with inflammatory receptors such as toll-like receptors 2 (TLR2) [13, 14] creating a vicious cycle of worsening inflammation and tau pathology [12-15]. Our data demonstrated that CK2α’ haploinsufficiency significantly reduced the number of Iba1 + cells in the hippocampus, reduced the levels of the microglia reactive marker CD68, ameliorated microglia morphological changes associated with pathology, reduced the levels of pro-inflammatory cytokines in PS19 mice and reduced microglia phagocytic activity. Previous studies in human microglia derived from hiPSCs treated with CK2 inhibitors reduced cytokines production [99]. Therefore, all together, these data support a specific key role of CK2α’ in mediating neuroinflammation via activation of microglia.

Loss of synaptic density and neuronal dysfunction has been previously connected to increased phagocytic microglia [64, 89, 108, 109]. The activation of the Complement Component pathway in microglia is associated with enhanced phagocytic activity. While such activation is beneficial to clear Tau aggregates and get rid of dysfunctional synapses, chronic activation results in excessive pruning and an aberrant loss of synapses [80, 89]. Our results demonstrated a decrease in microglia phagocytosis of the PSD95 synaptic marker in PS19;CK2α’^(+/−)^ mice suggesting that CK2α’ is influencing the phagocytosis of synapses by microglia, but the mechanisms behind this are unclear. Complement component 1q (C1q) is often seen at synapses that accumulate pTau, and the levels of C1q are associated with enhanced phagocytic microglia engulfment of synapses leading to the decline of synapse density, synaptic function and cognitive decline associated with AD[89]. In line with these previous findings, our RNA-seq data revealed the upregulation of genes related to synaptic pruning in PS19 mice, especially those associated with C1q. Importantly, antibodies targeting C1q have rescued synapse loss in tauopathy models[89]. While manipulation of CK2α’ did not impact the expression of C1q, it is possible that CK2α’ is impacting the complement pathway via phosphorylation of critical components, such as Complement component 9 (C9) and complement component 1r (C1r), which are known targets of CK2 [110, 111]. Further studies focusing on the impact of CK2α’ on the classical cascade mediated synapse engulfment will be important to elucidate the mechanisms involved in improved synaptic density, function, and cognition.

An intriguing finding from our study is the apparent discrepancy between the upregulation of genes associated with immune and innate responses and the reduction of neuroinflammatory phenotypes in microglia in PS19;CK2α’^(+/−)^ mice. Typically, the transcriptional activation of innate immune pathways is linked to pathology and neuroinflammation. In PS19 mice, such activation, along with increased expression of genes related to microglial dysfunction and reactivity, has been extensively reported [80, 81]. Surprisingly, while CK2α’ haploinsufficiency further amplified this transcriptional immune response in PS19 mice, we observed a concomitant reduction in neuroinflammatory markers, accompanied by improvements in neuronal function and behavior. This apparent paradox may reflect a primed but non-pathological immune state, the engagement of anti-inflammatory regulatory mechanisms, or a temporal or cell-type-specific decoupling between transcriptional signatures and functional outcomes. Similar findings have been reported in studies involving partial agonists of the tropomyosin receptor kinase B (TrkB) and C (TrkC), where synaptic deficits were rescued in the APP model of AD despite enhanced expression of microglial neuroinflammatory markers and immune responses typically associated with late-stage pathology [112]. Additionally another study reported that INPP5D haploinsufficiency in the PS19 mouse resulted in decreased tau pathology and ameliorated motor deficits [113], yet increased inflammatory signatures seen via RNA-Seq. Collectively, these observations underscore the nuanced role of immune signaling in the CNS and caution against interpreting transcriptional evidence of inflammation as a direct indicator of detrimental neuroimmune activation.

## Conclusions

In conclusion, our findings identify CK2α’ as a central and previously underappreciated regulator of tauopathy pathogenesis, acting at the crossroads of immune signaling, synaptic function, and tau phosphorylation. By demonstrating that CK2α’ haploinsufficiency alleviates tau pathology, reduces neuroinflammation, and improves synaptic integrity and function in PS19 mice, we provide compelling genetic evidence that CK2α’ contributes to multiple pathological hallmarks of tau-driven neurodegeneration. Importantly, these effects appear to occur through complex, context-dependent mechanisms that may differ across cell types and disease stages. The observation that CK2α’ influences both neuronal and microglial compartments underscore the need to dissect its cell-autonomous versus non-cell-autonomous roles. Given the growing interest in CK2 as a therapeutic target and the limitations of current inhibitors that lack subunit specificity, the development of CK2α’-selective modulators [114] may represent a promising avenue for disease-modifying therapies in tauopathies and related neurodegenerative conditions. Future studies elucidating the molecular and cellular pathways downstream of CK2α’ will be critical for translating these findings into clinical applications.

## Abbreviations

AD: Alzheimer's Disease
ADRD: Alzheimer's Disease and Related Dementias
APP: Amyloid Precursor Protein
Aβ: Amyloid β
C1q: Complement component 1q
C1r: Complement component 1r
C9: Complement component 9
DGE: Differential gene expression
EC: Entorhinal cortex
fEPSP: Field excitatory postsynaptic potentials
FTD: Frontotemporal Dementia
GSVA: Gene set variation analysis
HD: Huntington’s Disease
HIP: Hippocampus
hTau: Human Tau
I2PP2a/SET: Inhibitor of protein phosphatase 2a inhibitor/SET
LTP: Long term potentiation
MAPT: Microtubule associate protein Tau
N2a: Neuro-2a
OL: Occipital cortex
PCx: Parietal cortex
PFC: Prefrontal cortex
PPF: Paired-pulse faciliation
PSD95: Post-synaptic density 95
pTau: Phosphorylated Tau
ScrRNA: Scrambled RNA
SFG: Superior frontal gyrus
siCK2α: Silencing RNA for CK2α
siCK2α’: Silencing RNA for CK2α’
snRNA-Seq: Single nucleus RNA sequencing
STP: Short term potentiation
TBI: Traumatic brain injury
TBS: Theta burst stimulation
TCx: Temporal cortex
TrkB: **Tropomyosin receptor kinase B**
TrkC: **Tropomyosin receptor kinase C**
Vglut1: Vesicular glutamate transporter 1
